# PIM kinase inhibition attenuates pro-tumoral and immunosuppressive functions of macrophages in classic Hodgkin lymphoma

**DOI:** 10.1038/s41419-025-08402-5

**Published:** 2025-12-26

**Authors:** Maciej Szydłowski, Ewa Kurtz, Filip Garbicz, Julia Maroszek, Michał Pawlak, Natalia Ochocka, Marcin Tabaka, Monika Prochorec-Sobieszek, Anna Szumera-Ciećkiewicz, Patryk Górniak, Olga Szymańska-Giemza, Grzegorz Rymkiewicz, Agnieszka Kołkowska-Leśniak, Wojciech Kukwa, Ewa Paszkiewicz-Kozik, Justyna Totoń-Żurańska, Sylwia Radomska, Zofia Pilch, Dominika Nowis, Jakub Golab, Michał Kurlapski, Jan Maciej Zaucha, Alicja Braczko, Marcin Kaszkowiak, Paweł Wołkow, Katarzyna Wiktorska, John Brognard, Sabina Lichołai, Ewa Lech-Marańda, Przemysław Juszczyński

**Affiliations:** 1 Department of Experimental Hematology, Institute of Hematology and Transfusion Medicine, Warsaw, Poland; 2 Centre of Postgraduate Medical Education, Doctoral School, Warsaw, Poland; 3 Computational Genomics Group, International Centre for Translational Eye Research – ICTER, Warsaw, Poland; 4 Institute of Physical Chemistry, Polish Academy of Sciences, Warsaw, Poland; 5 Department of Diagnostic Hematology, Institute of Hematology and Transfusion Medicine, Warsaw, Poland; 6 Biobank, Maria Sklodowska-Curie National Research Institute of Oncology, Warsaw, Poland; 7 Flow Cytometry Laboratory, Dept. of Cancer Pathomorphology, Maria Sklodowska-Curie National Research Institute of Oncology, Warsaw, Poland; 8 Department of Hematology, Institute of Hematology and Transfusion Medicine, Warsaw, Poland; 9 Department of Otolaryngology, Faculty of Dental Medicine, Medical University of Warsaw, Warsaw, Poland; 10 Department of Lymphoid Malignancies, Maria Sklodowska-Curie National Research Institute of Oncology, Warsaw, Poland; 11 Center for Medical Genomics OMICRON, Jagiellonian University Medical College, Cracow, Poland; 12 Department of Immunology, Medical University of Warsaw, Warsaw, Poland; 13 Laboratory of Experimental Medicine, Medical University of Warsaw, Warsaw, Poland; 14 Department of Hematology and Transplantology, Medical University of Gdansk, Gdansk, Poland; 15 Department of Biochemistry, Medical University of Gdansk, Gdansk, Poland; 16 Broad Institute of Massachusetts Institute of Technology and Harvard, Cambridge, MA USA; 17 Division of Laboratory Diagnostics and Clinical Epigenetics, University of Rzeszów, Medical College, Faculty of Medicine, Institute of Medical Sciences, Rzeszów, Poland; 18 Department of Physics and Biophysics, Institute of Biology, Warsaw University of Life Sciences-SGGW, Warsaw, Poland; 19 Laboratory of Cell and Developmental Signaling, National Cancer Institute, NIH, Frederick, MD USA

**Keywords:** Experimental models of disease, Hodgkin lymphoma

## Abstract

Tumor-associated macrophages (TAMs) of classic Hodgkin Lymphoma (cHL) contribute to the development of immunosuppressive tumor microenvironment (TME) and are associated with worse treatment outcomes. However, detailed features, functions and therapeutic vulnerabilities of cHL TAMs remain largely unknown. To address this, we analyzed cHL diagnostic biopsies by Cellular Indexing of Transcriptomes and Epitopes by Sequencing (CITE-seq) and assessed transcriptional, proteomic and metabolic profiles of in vitro TAM models. We show that Reed–Sternberg (RS) cells induce a disease-specific TAM phenotype, characterized by elevated expression of factors involved in chemotaxis, angiogenesis, extracellular matrix remodeling and tumor immune escape. RS cell-conditioned TAMs expressed TGFβ, CCL17 and tryptophan catabolizing enzymes, IDO1 and IL4I1, promoting regulatory T cell recruitment and activation. In addition, we identified the expression of PIM1/2/3 kinases in cHL TAMs and characterized PIMs as critical hubs orchestrating RS-macrophage interactions. Pharmacological PIM blockade attenuated the RS-induced TAM transcriptional program. In established TAMs, PIM inhibition or PROTAC-mediated degradation decreased the expression of multiple factors associated with pro-tumoral TAM functions, including IL8, MMP9, CHI3L1/2, CD206, CD209, PD-L1, CCL17, TGFβ, IL4I1 and IDO1. PIM blockade attenuated TAM-dependent eosinophil chemoattraction, extracellular matrix remodeling, angiogenesis and regulatory T-cell development. Taken together, our study highlights the role of PIMs in the regulation of pathogenic TAM functions in cHL, further supporting the rationale of PIM targeting in this disease.

## Introduction

Classic Hodgkin lymphoma (cHL) is a B-cell malignancy characterized by pathognomonic Hodgkin/Reed-Sternberg (RS) cells embedded in extensive, but ineffective inflammatory/immune cell infiltrate. The lack of effective immune response in cHL kindled numerous studies that revealed various mechanisms utilized by RS cells to evade antitumor immunity, including CD274/PDCD1LG2 copy gains and increased expression of the PD-1 ligands, or LGALS1 (galectin-1) overexpression [1, 2]. Subsequent clinical trials demonstrated the clinical efficacy of PD-1 blockade in relapsed/refractory cHL patients [3, 4]. However, despite high response rates in cHL patients receiving PD-1 blockade, a fraction of patients are refractory or relapse after initial response [5], demonstrating an incomplete understanding of immune evasion mechanisms and the role of immunosuppressive tumor microenvironment (TME) in modulation of antitumor immunity in cHL. Furthermore, malignant RS cells exhibit frequent losses of MHC class I expression [6], which preclude antigen presentation to CD8^+^ T cells and CD8 T-cell-mediated tumor killing. These observations suggest a leading role of CD4^+^ T cells in PD-1 blockade-mediated antitumor responses [6–8]. Thus, understanding the mechanisms limiting CD4-mediated tumor immunity is crucial to develop more effective therapeutic strategies in cHL.

The cellular ecosystem of the cHL TME is thought to be shaped by a plethora of signals, initially derived from RS cells and subsequently co-evolving in the course of the disease. Tumor-associated macrophages (TAMs) are important components of the cHL TME, associated with inferior outcome, tumor immune escape and resistance to immunotherapies [9–11]. Several recent studies provided the first insights into gene expression profiles, TAM immunophenotype, and their spatial relations and functions in the cHL TME [12–16]. The malignant RS cells reside in close proximity to PD-1^+^CD4^+^ T cells and PD-L1^+^ macrophages, which form a localized immunoprotective niche [12]. These studies provided a biological rationale for the therapeutic targeting of myeloid cells in the cHL TME. However, given the complexity and redundancy of communication channels between tumor cells and macrophages, therapeutic interventions aiming at depletion or reprogramming TAMs were largely ineffective and a clinically effective TAM targeting strategy remains to be developed [17].

In our previous studies, we demonstrated that PIM serine/threonine kinases 1/2/3 (PIMs) were ubiquitously expressed in primary and cultured RS cells [18]. Genetic or chemical PIM inhibition induced RS cell apoptosis and decreased the expression of multiple molecules engaged in developing the immunosuppressive microenvironment, including PD-L1/2, galectin-1 and CSF-1. These studies indicated that PIM kinases in cHL exhibit pleiotropic effects, likely influencing the composition of the TME and orchestrating tumor immune escape.

In this study, we leveraged Cellular Indexing of Transcriptomes and Epitopes by Sequencing (CITE-seq) [19], combined with transcriptional, proteomic and metabolic analyses of primary TAMs and in vitro TAM models to comprehensively characterize their functions and identify targetable vulnerabilities. We demonstrate that PIMs are expressed not only by RS cells but also in primary cHL TAMs and in vitro-generated TAM models. Inhibition of PIM kinases attenuated chemoattraction of monocytes and development of cHL TAMs in vitro and in mice bearing RS cell xenografts. In developed and polarized cHL TAMs, PIM kinases augment their tumor-supporting and immunosuppressive characteristics. Inhibition of PIM kinases in cHL TAMs with a clinical-grade pan-PIM inhibitor, MEN1703 (dapolsertib, MEN), markedly decreased expression of mediators of angiogenesis, chemotaxis, extracellular matrix (ECM) remodeling and immunosuppression. As a consequence, PIM inhibition in TAMs decreased eosinophil chemoattraction, extracellular collagen uptake, vessel formation and induction of regulatory T cells (Tregs). Taken together, we demonstrate that PIM kinases are critical for RS-TAM communication and TAM programming. Inhibition of PIM kinases might represent a therapeutic strategy for blocking manifold tumor-promoting activities of TAMs in cHL.

## Materials and methods

Detailed description of materials and methods (cell lines, chemicals, macrophage differentiation/polarization, patient samples, RNA-seq and CITE-seq, phospho-protein arrays, flow cytometry, metabolic and functional assays, animal studies) is included in the supplemental document.

### Statistical analysis

Statistical analysis was done using GraphPad Prism software (GraphPad Software Inc., La Jolla, CA, USA). Appropriate statistical tests (Kolmogorov–Smirnov test, two-sided *t* test and one-way ANOVA test) were used based on data distribution and are indicated in figure legends. Differences with *P* < 0.05 were deemed significant. The *p*-values were marked with the asterisks (*p < 0.05; **p < 0.01;***p < 0.001). Data are presented as mean ± SD and are derived from the number of independent biological replicates in the experiment or mice, specified in the figure legends. No statistical method was used to predetermine sample size for animal studies; instead, the size was chosen empirically based on our prior experience with calculating experimental variability. In the in vivo studies, animals were randomized based on size of developed tumors to generate groups with equal average tumor sizes and investigators were not blinded to the group allocation during the experiment or when assessing the outcome.

## Results

### PIM kinases are expressed in tumor-associated macrophages in classical Hodgkin lymphoma

To characterize the TAMs in cHL TME and integrate gene expression and immunophenotype at single-cell resolution, we collected 9 classical Hodgkin lymphoma (cHL) biopsies from treatment-naïve patients (Supplementary Table 1) and performed CITE-Seq. Additionally, we included reactive tonsils (RLT, reactive lymphoid tissue) from 3 donors as a reference. After quality control, we obtained 9476 cells from cHL tissues and 5089 cells from RLT. Given the low fraction of myeloid cells in the final dataset (especially in the RLT samples), we integrated our data with an external RLT dataset derived from 6 donors to balance the number of myeloid cells in tumor samples and controls to ensure robust downstream analyses [20].

**Fig. 1:**
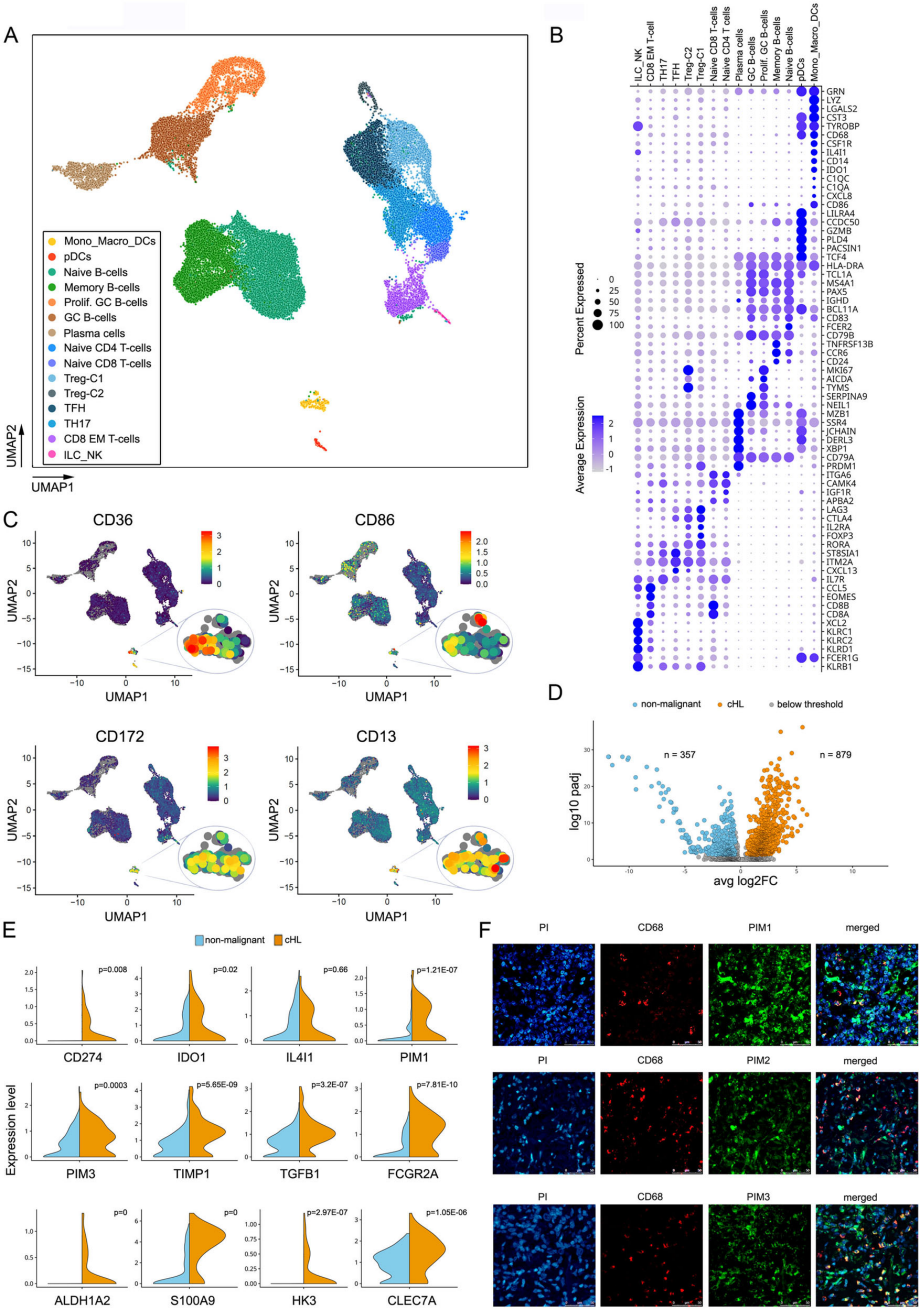
Expression profiles and immunophenotype of tumor-associated macrophages in cHL determined at single-cell resolution using CITE-seq. **A** Uniform Manifold Approximation and Projection (UMAP) plot of *n* = 42846 cells from an integrated scRNA-seq data set comprising cHL (*n* = 9476), RLT (*n* = 5089) and RLT cells derived from external source (*n* = 28281) cells, colored by cell type. Cluster signature genes are provided in Supplementary Table 2. **B** Expression of marker genes in identified cell clusters. Color intensity indicates average expression levels and dot size represents the percentage of cells expressing a marker gene in a given cluster. **C** Expression of macrophage/TAM-associated cell surface proteins. UMAP plots show antibody-derived signals for the indicated CITE-seq antibodies projected on the plot from (**A**). Color scale indicates measured expression levels. **D** Volcano plot showing differentially expressed genes between the cHL (orange) and RLN (blue) cells of the M/M/cDC cluster. Transcripts with an absolute log2 fold change (log2FC) greater than 1 and p-values less than 10^−5^ were considered significantly differentially expressed; transcripts outside these thresholds are indicated in gray. **E** Normalized expression of selected genes associated with TAM functions in M/M/cDC cluster. Orange indicates cHL, and blue represents RLN. Statistical differences in expression between the groups were assessed using Kolmogorov–Smirnov test. **F** Immunofluorescence staining for CD68 (red) and PIM1/2/3 kinases (green) shows expression of the kinases cHL tissue-derived macrophages. Blue represents propidium iodide (PI) counterstain.

Following gene expression and knowledge-based cell type annotation, we identified 15 cell type clusters, encompassing 4 B-cell clusters, plasma cells, 7 T-cell clusters, ILC/NK, pDC cells, and a myeloid cell cluster comprising monocytes, macrophages and conventional DCs (M/M/cDC cluster, Fig. 1A, B). While most of the identified immune cell clusters were present both in cHL and RLT (Supplementary Fig. S1A, B), we observed an increased abundance of T cells in cHL tissue, whereas certain other cell types (e.g. naïve B-cells, memory B-cells, proliferating B-cells and GC B-cells) were markedly underrepresented when compared to RLT (Supplementary Fig. S1B). M/M/DCs showed certain level of heterogeneity, marked by the expression of monocyte, macrophage, and dendritic cell-associated markers. Nonetheless, the transcriptomic features of the M/M/cDC cluster cells markedly resembled the previously described mononuclear phagocyte (MNP) cluster, comprising dendritic cells, monocytes and macrophages (Supplementary Fig. S1C) [14]. Transcriptomic profiles of the M/M/cDC and MNP were enriched in genes linked to phenotype and functions of tumor-associated macrophages (TAMs), including *CD68, CSF1R, CD14, IDO1, IL4I1* and *CD86* (Fig. 1B, Supplementary Fig. S1D) [10, 16–20]. In addition, the M/M/DCs showed exclusive expression of macrophage protein markers including CD36, CD86, CD172 (SIRP1α) and CD13, thus confirming the macrophage signature at both transcriptomic and protein level (Fig. 1C, Supplementary Fig. S1E) [10, 21–23].

Having confirmed the identity of the cells in the M/M/cDC “macrophage” cluster, we next compared the gene expression levels between cHL M/M/cDCs and normal RLT macrophages. We identified 879 genes with higher expression and 357 genes expressed at lower levels in cHL macrophages (Fig. 1D). Consistent with previous reports [13, 14], cHL macrophages demonstrated elevated mRNA levels of CD274 and IDO1 (Fig. 1E). In addition, we found increased expression of multiple M2-macrophage-associated genes: L-amino-acid oxidase - IL4I1 (IL4-induced 1), TIMP1, TGFB1, FCGR2A, ALDH1A2, S100A9, HK3, CLEC7A [21]. Interestingly, compared to macrophages in RLT, cHL TAMs exhibited higher expression of PIM-1 and -3 kinases, (Fig. 1E). Since our previous studies demonstrated that PIM kinases are expressed by RS cells and facilitate their immune escape [18], we specifically assessed the expression of PIM kinases (PIM1, -2 and -3) in CD68^+^ macrophages present in cHL tissue using immunofluorescence microscopy (Fig. 1F). Consistent with the scRNA-seq data, these analyses confirmed the expression of PIM1/2/3 in CD68^+^ TAMs, raising further questions about PIM kinase functions in these cells.

### Development and characterization of cHL TAM in vitro model

**Fig. 2:**
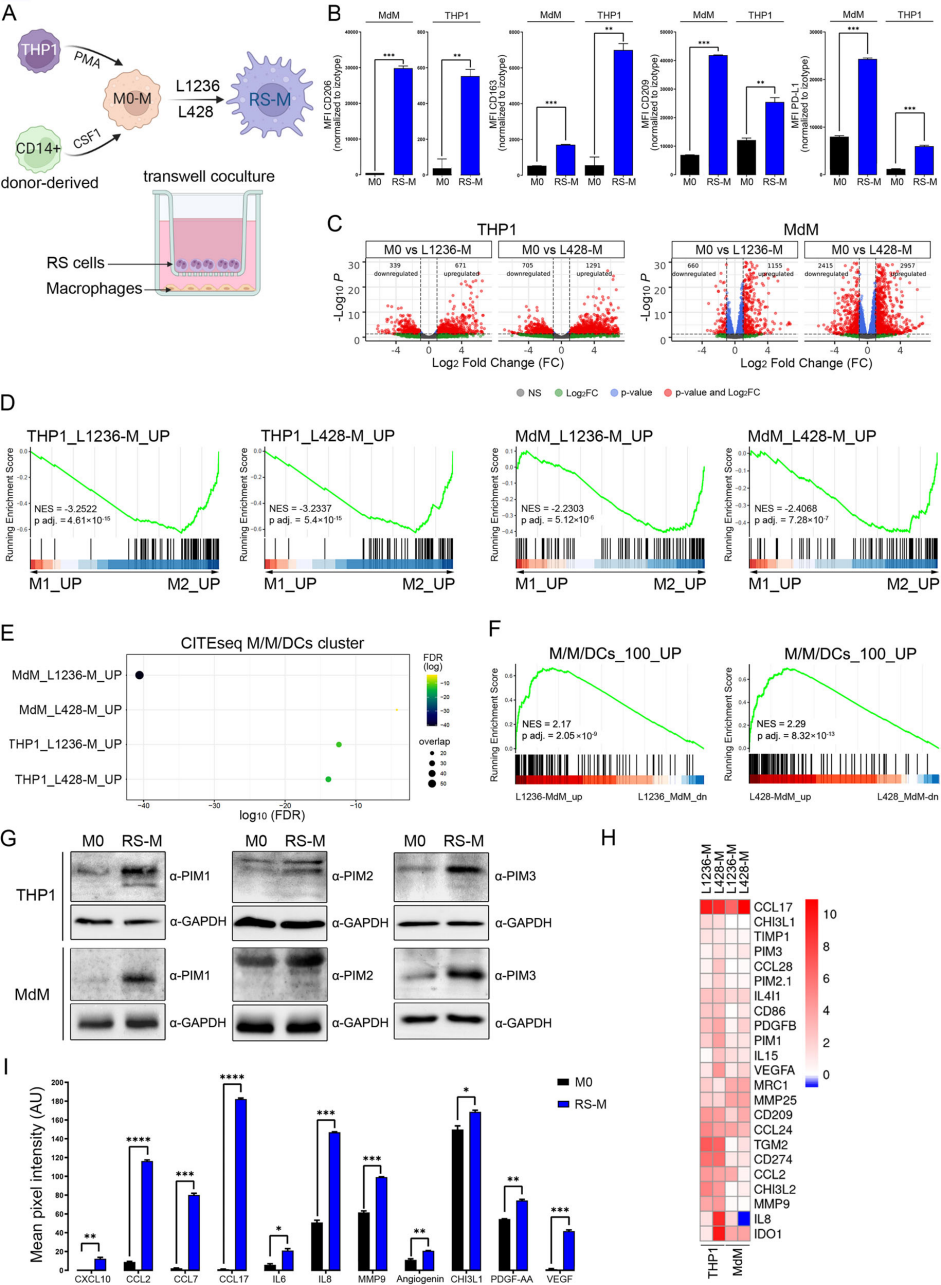
Detailed characterization of tumor-associated macrophages in classic Hodgkin lymphoma. **A** A diagram illustrating the generation of the in vitro models for TAMs in cHL. THP1 cells and healthy donor-derived monocytes were differentiated into macrophages (M0-M) using PMA and CSF-1, respectively. To generate RS-educated macrophages (RS-M), M0 cells were cocultured for five days with L428 or L1236 RS cells under conditions precluding direct contacts. **B** Immunophenotype of RS-M generated from monocyte-derived-macrophages (MdM) and THP1 cells. Surface expression of CD206, CD163, CD209 and PD-L1 in M0 and RS-M was determined by flow cytometry. Median fluorescence intensity (MFI) values of indicated antibodies were normalized to isotype control. Error bars indicate ±SD of two independent biological replicates in a representative experiment. *P*-values were determined using the two-sided *t*-test: **p < 0.01; ***p < 0.001. **C** Volcano plots with differentially expressed genes in the M0 cells and RS-conditioned macrophages (RS-M). RS-M were generated from THP1 cells or normal monocytes (MdM, monocyte-derived macrophages) using L1236 and L428 cell lines (L1236-M and L428-M, respectively). Transcripts marked in red indicate significant expression differences (absolute log2 fold change (log2FC) greater than 1 and *p*-values less than 0.05). **D** Gene set enrichment analysis of genes upregulated in THP1 and monocyte-derived macrophages (MdM) conditioned by L1236 or L428 cells in the space of M1 and M2 signatures. Top 250 genes upregulated in L1236 or L428-conditioned THP1-M (THP1_L428-M_UP, THP1_L1236-M_UP) or MdMs (MdM_L428-M_UP, MdM_L1236-M_UP) were projected on ordered M1 vs M2 signature (M1_UP and M2_UP) identified by Gerrick et al. [21]. Respective macrophage-upregulated gene set names are indicated above the GSEA plots. Black bars represent the position of the THP1 or monocyte-derived macrophage genes in the ranked and fold-change ordered list of M1 vs M2 transcripts, together with the running enrichment score (green line). NES: normalized enrichment; p.adj: BH-corrected *p*-value. L1236 and L428 THP1 and MdM signatures are provided in Supplementary Table 3. **E** Hyper-geometric enrichment analysis showing overlapping transcriptional signatures of M/M/cDC cluster and THP1- or monocyte-derived macrophages induced by L1236 and L428 cells. Top 250 genes upregulated in L1236 or L428-conditioned THP1-M or MdMs (Supplementary Table 3) were used to calculate the hyper-geometric enrichment. Color scale indicates the false discovery rate (FDR), dot size represents the number of overlapping genes. **F** Gene set enrichment analysis of top 100 upregulated genes of M/M/cDC cluster in the ranked transcriptional signature of L1236 or L428-induced MdMs. Black bars represent the position of the M/M/cDC signature genes in the ranked and fold-change ordered list of transcripts up- or downregulated in L1236 or L428-induced MdMs. The green line indicates the running enrichment score. NES: normalized enrichment; p.adj: BH-corrected *p*-value. **G** Immunoblot analysis of PIM1, PIM2 and PIM3 expression in M0 and RS-conditioned MdM and THP1 cells. GAPDH served as a loading control. Blots are representative of three independent experiments. Immunoblot images have been cropped for presentation. Uncropped immunoblot images are provided in the Supplemental Material. **H** Expression of M2/TAM-associated genes in RS-conditioned, monocyte or THP1-derived macrophages. The color scale indicates transcript abundance relative to M0 macrophages (blue – lower, red – higher than in M0; values next to the color scale indicate log2 fold change). **I** Cytokine secretion profile of RS-conditioned MdMs. Expression levels of indicated cytokines were estimated from duplicates in a Proteome profiler Human XL cytokine array kit by measuring mean pixel intensity using ImageJ software. Corresponding raw data are shown in Supplementary Fig. S2D. Error bars represent the ±SD of two independent replicates. *P*-values were determined using the two-sided *t* test: *p < 0.05; **p < 0.01; ***p < 0.001.

Since characterization and functional studies of primary TAMs are not feasible, we first developed in vitro TAM models. To mimic paracrine interactions of RS cells with macrophages in TME suggested in previous studies [15, 16], we created a coculture system in which THP1 or monocyte-derived macrophages were co-incubated with RS cell lines (L1236 or L428) under conditions precluding direct cell-to-cell contacts (Fig. 2A). After 5 days of coculture, RS-polarized macrophages (RS-M) were subjected to immunophenotyping and gene expression profiling by RNA-seq. Coculture with L1236 RS cells induced surface expression of TAM-linked CD163, CD206, CD209 and PD-L1 (Fig. 2B) and was associated with significant transcriptional changes (Fig. 2C). To characterize our model cells in an unbiased manner, we assessed a global transcriptional similarity between model macrophages (derived from normal peripheral blood monocytes or from a monocytoid cell line THP1), and the classically (LPS + IFNγ) or alternatively (IL4 + IL13) – activated macrophages (“M1” or “M2”-like) [21]. The transcriptional profiles of RS-M generated using both L1236 and L428 RS cells exhibited features typical for alternatively- activated macrophages (“M2”-like) (Fig. 2D). Genes upregulated in macrophages by RS cells were associated with TGFβ1, IL4, IL10, IL13 and IL6 pathways via JAK-STAT signaling, as revealed by GSEA (Supplementary Fig. S2A). Importantly, RS-conditioned, THP1 or monocyte-derived macrophages upregulated an inflammatory signature linked to IL-1β signaling, recently reported to be associated with inferior response to immune checkpoint blockade [22] (Supplementary Fig. S2B, C). Next, to determine whether the RS-conditioned macrophages are a representative model of primary cHL-associated macrophages in the tumor tissue, we compared transcriptional signatures of RS-M and M/M/cDC cluster cells identified in CITE-seq. As determined in enrichment analyses, the M/M/cDC cluster signature significantly overlapped with genes upregulated in macrophages by RS cells (Fig. 2E). In addition, genes induced by RS cells in macrophages in vitro were preferentially enriched in the M/M/cDC cluster cells (Fig. 2F). In line with the results of the CITE-seq and immunofluorescence microscopy analyses, RS-M exhibited elevated protein levels of PIM kinases (Fig. 2G).

To further characterize these RS-conditioned macrophages, we assessed the activity of signaling pathways and cytokine/chemokine secretion by RS-M cells using phospho-kinase and cytokine protein arrays, respectively. Given the highly similar transcriptional signatures of RS-M generated using both L1236 and L428 RS cell lines, we chose macrophages obtained in coculture with L1236 cells for these studies (Fig. 2H). Interaction with RS cells stimulated macrophages to produce chemokines/cytokines involved in shaping cHL TME (CCL2/MCP1, CCL7/MCP3, CCL17/TARC, CCL24/Eotaxin-2, CCL28, IL6), matrix remodeling (MMP9, −19, −25) and certain proangiogenic factors (Angiogenin, CHI3L1, PDGF-AA, VEGF, IL8) (Fig. 2H, I, Supplementary Fig. S2D). Importantly, most of these transcripts were also present in primary TAMs in our CITE–seq dataset, showcasing the relevance of the developed in vitro TAM model (Supplementary Fig. S2E). RS-M conditioned medium contained elevated levels of TIM3, an inhibitory molecule expressed by MNPs in cHL TME (Supplementary Fig. S2D) [14]. In addition to the observed changes in gene transcription, pathway activation and protein secretion, macrophages cocultured with RS cells underwent substantial metabolic reprogramming and exhibited enhanced glycolysis and mitochondrial respiration, compared to unpolarized, M0 macrophages (Supplementary Fig. S2F). Furthermore, TAMs developing in the presence of RS cells markedly upregulated the activity of M2-associated transcription factors: CREB1, STAT3, STAT6 and to a lesser extent, M1-associated STAT1 and cJun (Supplementary Fig. S2G, H) [23–25]. Taken together, these data indicate that the developed TAM model faithfully recapitulates primary cHL-associated TAM features and is appropriate for further functional studies.

### PIM kinases are involved in the development of the TAM phenotype induced by RS cells

**Fig. 3:**
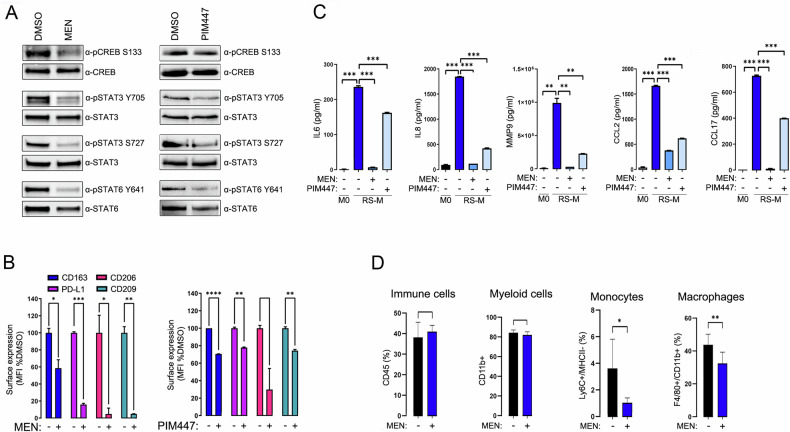
PIM inhibition during macrophage polarization attenuates the acquisition of TAM features induced by RS cells. **A** PIM inhibition attenuates the activity of M2-associated factors in macrophages cocultured with RS cells. After 5-day coculture with RS cells (under conditions precluding direct cell-to-cell contacts) in the presence or absence of 3 µmol/L MEN or 7 µmol/L PIM447, THP1-derived macrophages were harvested and lysed. Phosphorylation levels of CREB (S133), STAT3 (S705 and S727) and STAT6 (Y641) and the corresponding total protein levels were assessed by immunoblotting. Data are representative of three independent experiments. Immunoblot images have been cropped for presentation. Uncropped immunoblot images are provided in the Supplemental Material. **B** PIM inhibitors block the development of specific RS-M immunophenotype. THP1-derived macrophages were cocultured with RS cells in the absence (−) or presence (+) of PIM inhibitors and surface expression of CD163, PD-L1, CD206 and CD209 was assessed by flow cytometry. Median fluorescence intensity (MFI) values of indicated antibodies were normalized to isotype control. Error bars indicate ±SD of two independent replicates in a representative experiment. *P*-values were determined using the two-sided *t*-test: **p < 0.01; ***p < 0.001. Data are representative of three independent experiments. **C** PIM blockade attenuates the secretion of cytokines, chemokines and matrix remodeling proteins induced by RS cells. RS-conditioned macrophages cultured in the presence (+) or absence (−) of the pan-PIM inhibitors were harvested and seeded in equal numbers. After 24 h, levels of IL6, IL8, MMP9, CCL2 and CCL17 in cell culture supernatants were determined using bead-based immunoassay and flow cytometry. M0 macrophages were used as a reference. Bars represent mean values ± SD of two independent replicates in a representative experiment. *P*-values were determined using the two-sided *t*-test: **p < 0.01; ***p < 0.001. Data are representative of three independent experiments. **D** PIM inhibition decreases the abundance of monocytes and macrophages in mice bearing RS cell xenograft tumors. NSG mice (*n* = 12) were inoculated with L428 RS cells and treated with MEN (75 mg/kg) or H_2_O (vehicle) for 21 days (*n* = 6 per group). After treatment, tumors were explanted and cells were enumerated by flow cytometry.

Since at least part of the M2-associated pathways and transcription factors induced in RS-educated TAMs (e.g., STAT3 and CREB1) are regulated by PIM kinases [26–28], we hypothesized that induction and activation of PIMs in monocytes/macrophages following RS cell interaction might facilitate macrophage polarization and acquisition of TAM phenotype and transcriptional profile. To test this hypothesis, we polarized RS-M as described above, but in the presence of a clinical-grade pan-PIM inhibitor, MEN in the medium and assessed the activity of STATs and CREB1 transcription factors in obtained TAMs. MEN blocked phosphorylation of CREB1(S133), STAT3 (Y705 and S727) and STAT6 (Y641) (Fig. 3A). In addition, MEN attenuated induction of CD163, CD206, CD209 and PD-L1 expression on the surface of macrophages cocultured with RS cells (Fig. 3B) and impaired secretion of IL-6, IL-8, CCL2, CCL17 and MMP9 (Fig. 3C). Similar effects, although less marked, were observed using additional, structurally unrelated pan-PIM inhibitor – PIM447. These findings indicate that PIM inhibition during RS-macrophage cross-talk broadly inhibits acquisition of the typical cHL TAM phenotype and function.

### PIM inhibition decreases monocyte/macrophage chemoattraction to the tumor site in the RS xenograft model

We and others have previously demonstrated that RS cells express the crucial macrophage differentiation and polarization factor - CSF1, a monocyte chemoattractant CCL5, and an M2-polarizing factor, IL13 [18]. Since RS-Ms exhibited increased expression of multiple chemokines attracting monocytes/macrophages (CCL2/MCP1, CCL7/MCP3, CXCL10, Fig. 2H, I, Supplementary Fig. S2D), we hypothesized that their secretion perpetuates myeloid cell infiltration via self-reinforcing positive feedback loop, initiated by RS cells. Of note, expression of these chemokines/cytokines in RS-M and RS cells was markedly attenuated by the PIM inhibitor (Fig. 3C, Supplementary Fig. S2I and ref. [18]). These observations prompted us to hypothesize that PIM inhibition would decrease monocyte/macrophage infiltration to the tumor site. To test this hypothesis, we established L428 tumors in NSG mice, treated the animals with MEN or vehicle, and enumerated tumor-infiltrating murine monocytes/macrophages using flow cytometry. Although the total immune cell content did not differ between both groups (control and MEN-treated), monocytes and macrophages were significantly less abundant in tumors explanted from MEN-treated mice, compared to tumors from control animals (Fig. 3D). These data indicate that PIM kinases support monocyte/macrophage chemoattraction and RS-mediated TAM programming in cHL.

### PIMs sustain pro-tumoral phenotype of macrophages in cHL

**Fig. 4:**
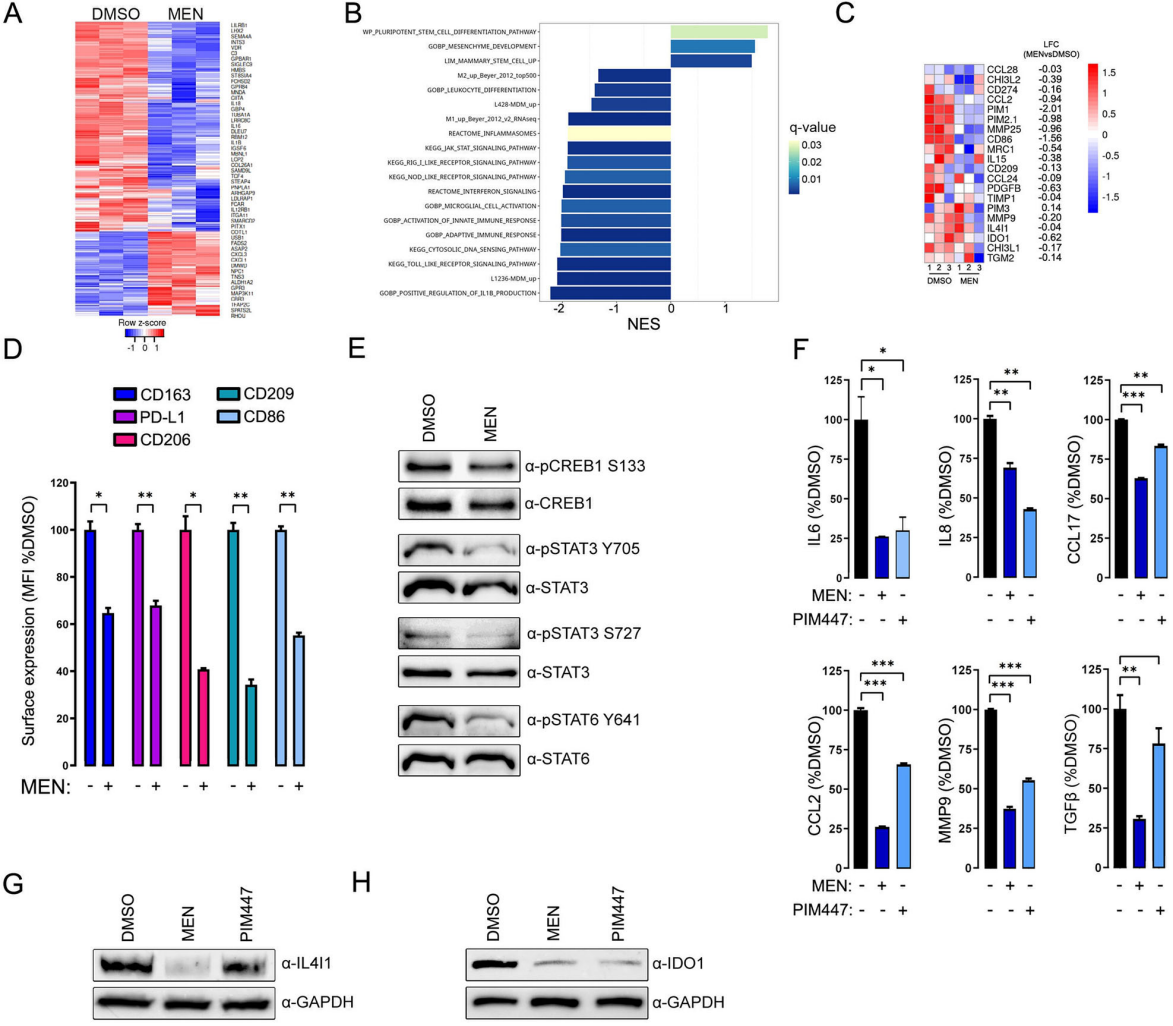
PIM inhibition targets the transcriptional program and the phenotype of macrophages acquired through their interactions with Reed–Sternberg cells. **A** PIM inhibition decreases expression of L1236-MdM signature genes. L1236-conditioned THP1-derived macrophages were treated with DMSO or 3 µmol/L MEN for 24 h and subjected to gene expression profiling by RNA seq. The heat map indicates expression of L1236-MdM signature genes (determined as in Fig. 2C, D) in L1236-THP1 macrophages after PIM inhibition. Please refer to Supplementary Fig. S3A for GSEA quantification. **B** PIM inhibitor-induced changes in gene set expression in L1236-conditioned THP1 macrophages. L1236-conditioned macrophages were treated with DMSO or 3 µmol/L MEN for 24 h (as in **A**) and subjected to gene expression profiling by RNA-seq. Genes up -or downregulated in MEN-treated cells were ranked by fold change and used to estimate the enrichment of MSigDB gene sets. Gene sets associated with cell differentiation, immune activation and response, inflammasomes, IFN, JAK/STAT and IL1β signaling are shown. Coordinated changes in genes upregulated in L1236- and L428-MdM are also shown. NES: normalized enrichment score; bar color indicates FDR *q*-value. Please refer to Supplementary Fig. S3B for heat maps demonstrating decreased expression of inflammasome/IL1β-associated gene sets. **C** Heat map showing expression of M2/TAM-associated genes in L1236-conditioned macrophages following exposure to MEN (as described in (**A**, **B**)). Color scale indicates relative gene expression levels. LFC: log fold change of MEN-treated macrophages relative to DMSO-treated cells. **D** PIM blockade modulates the immunophenotype of RS-conditioned macrophages. THP1-derived RS-M were treated with DMSO or MEN as in (**A**). Surface expression of CD163, PD-L1, CD206, CD209 and CD86 was assessed by flow cytometry. Bar graphs depict the average MFI (±SD) of indicated antibodies of two independent replicates in a representative experiment, normalized to isotype control and relative to DMSO treatment. *P*-values were determined using the two-sided *t*-test: *p < 0.05; **p < 0.01. Data are representative of three independent experiments. **E** Inhibition of PIM kinases in RS-conditioned macrophages decreases activity transcription factors associated with M2-macrophage polarization. THP1-derived RS-M were treated with MEN as in (**A**), harvested and lysed. Phosphorylation levels of CREB (S133), STAT3 (Y705 and S727), STAT6 (Y641) and the corresponding total protein levels were assessed by immunoblotting. Data are representative of three independent experiments. Immunoblot images have been cropped for presentation. Uncropped immunoblot images are provided in the Supplemental Material. **F** PIM blockade in RS-conditioned macrophages impairs the secretion of cytokines and chemokines. THP1-derived RS-M were treated with DMSO, 3 µmol/L MEN or 7 µmol/L PIM447 for 24 h. Following treatment, macrophages were collected washed and resuspended in a fresh medium in equal numbers for 24 h. Quantitative assessment of IL6, IL8, CCL17, CCL2 and MMP9 in culture supernatants was performed by flow cytometry. Bar graphs present average expression levels (±SD, whiskers) of two independent replicates in a representative experiment relative to DMSO treatment. *P*-values were determined using the two-sided *t*-test: *p < 0.05; **p < 0.01. Data are representative of three independent experiments. **G**, **H** Inhibition of PIM kinases downregulates IL4I1 and IDO1 expression. THP1-derived RS-M were treated with pan-PIM inhibitors as described in (**F**), harvested and lysed. IL4I1 and IDO1 protein levels were assessed by immunoblotting. GAPDH served as a loading control. Data are representative of three independent experiments. Immunoblot images have been cropped for presentation. Uncropped immunoblot images are provided in the Supplemental Material.

After demonstrating that PIM kinases facilitate the polarization of macrophages to pro-tumoral TAMs, we asked whether PIM inhibition would attenuate tumor-supportive and immunosuppressive features of already polarized TAMs. To address that question, we first characterized transcriptomic, immunophenotypic, biochemical and metabolic features of RS-educated TAMs following separation from RS cells and after MEN treatment. The PIM inhibitor decreased mRNA levels of genes upregulated by their interactions with RS cells (Fig. 4A, B, Supplementary Fig. S3A), suggesting that PIM blockade “reverses” the transcriptomic features of TAMs, acquired during RS-mediated programming. Among downregulated genes were those involved in the innate immune response, NOD- and RIG-I inflammasomes, IL-1β processing and production, angiogenesis, TME remodeling, chemoattraction, cell activation and differentiation (Fig. 4B, C, Supplementary Fig. S3B). Surprisingly, MEN-treated RS-M exhibited increased gene expression signatures associated with less differentiated cells, suggesting PIM involvement in both macrophage differentiation and polarization (Fig. 4B). Pharmacological PIM blockade in RS-M was associated with decreased surface levels of CD163, PD-L1, CD206, CD209 and CD86 (Fig. 4D, Supplementary Fig. S3C), and attenuated activity of CREB, STAT3 and STAT6 (Fig. 4E and Supplementary Fig. S3D). These effects were confirmed using a PIM-specific proteolysis-targeting chimaera (PROTAC), derived from the pan-PIM inhibitor SGI-1776 (SGI-VHL02) [29] (Supplementary Fig. S3E, F). Consistent with decreased expression of genes involved in cell activation, PIM inhibition dampened glycolysis, mitochondrial respiration and attenuated secretion of IL6, CCL17, IL8, CCL2 and MMP9 by RS-M (Fig. 4F, Supplementary Fig. S3G). In addition, MEN and PIM447 markedly decreased expression and secretion levels of TGFβ, IDO1 and IL4I1, a cytokine and two tryptophan-catabolizing enzymes facilitating Treg polarization at the tumor site, (Fig. 4F–H) [30–32]. Taken together, these data highlight the role of PIM kinases in regulating disease-specific programming of macrophages in cHL and their tumor-supportive functions (pro-angiogenic, TME remodeling and immunomodulatory).

### PIM inhibition decreases eosinophil chemoattraction, ECM remodeling and proangiogenic potential of TAMs

**Fig. 5:**
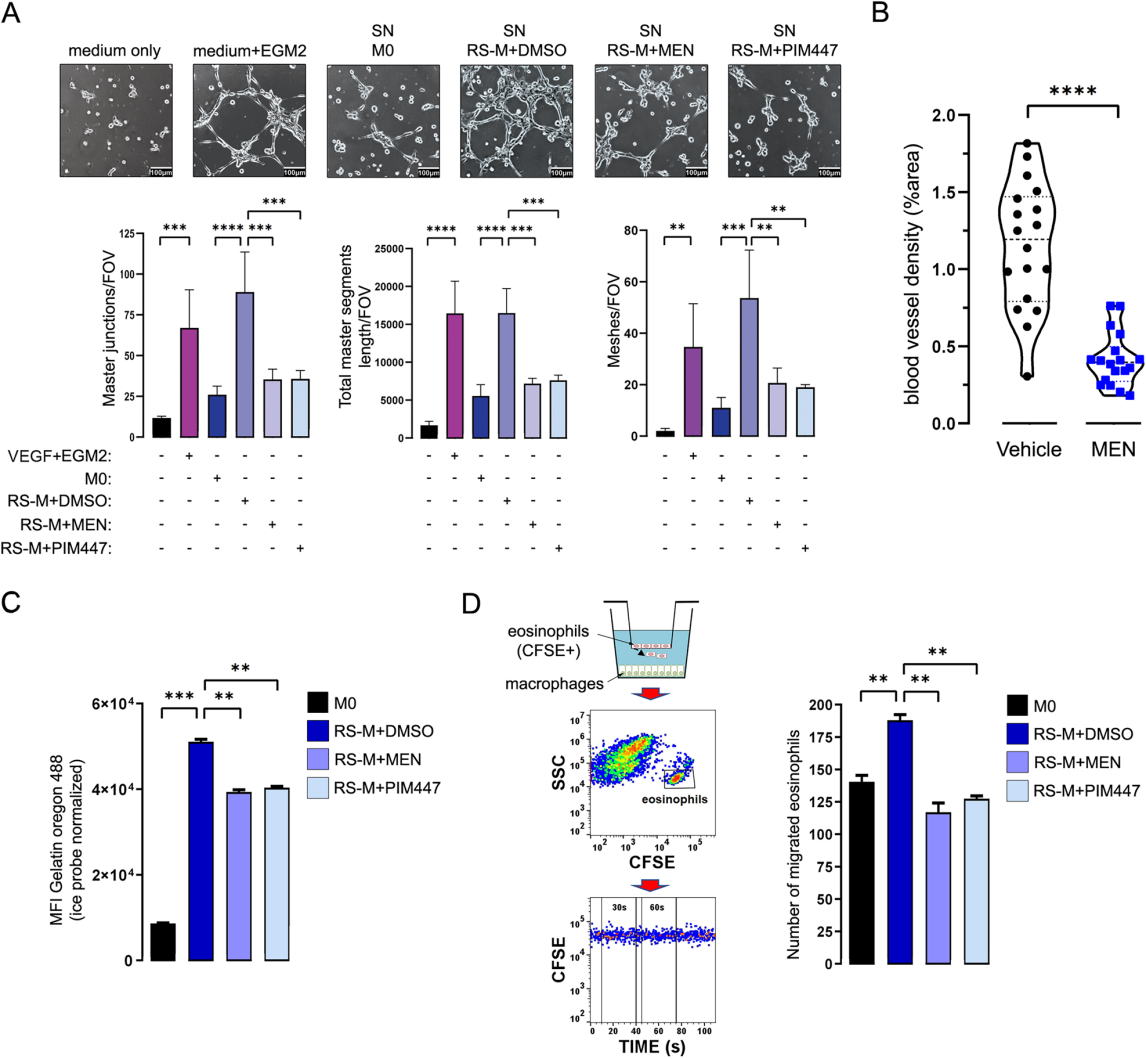
PIM inhibition attenuates pro-tumoral functions of TAMs. **A** Pan-PIM inhibitors attenuate the capacity of RS-conditioned macrophages to stimulate angiogenesis in vitro. THP1-derived, RS-conditioned macrophages (RS-M) were exposed to DMSO, 3 µmol/L MEN or 7 µmol/L PIM447 for 24 h. M0 and treated RS-M were collected and seeded in equal numbers in an unsupplemented EBM2 medium. Tube formation by HUVECs after stimulation with M0 and treated/untreated RS-M culture supernatants (SN) was evaluated after 24 h. Bar graphs below show the quantification of tube formation analysis performed using the Angiogenesis Analyzer plugin for ImageJ. Error bars indicate standard deviations of three independent fields analyzed per condition. One-way ANOVA with Šídák’s multiple comparisons test was used to determine statistical significance: **p < 0.01; ***p < 0.001; ****p < 0.0001. Each micrograph (captured at 10× magnification) shows a representative experiment’s result. Negative control (HUVECs stimulated with basal, non-supplemented EBM2 medium) and positive control (HUVECs stimulated with EMB2 medium with VEGF-A containing EGM2 supplement cocktail) are shown on the left. Scale bars: 100 μm. Data are representative of three independent experiments. **B** MEN attenuates tumor vessel formation in vivo. NSG mice (*n* = 12) were inoculated with L428 RS cells and treated with MEN (75 mg/kg) or H_2_O (vehicle) for 21 days (*n* = 6 per group). Blood vessel formation in harvested tumor sections was determined by immunohistochemistry and CD34 staining. Blood vessel density was quantified using a vessel analysis plug-in and ImageJ software. Three independent fields per tumor were used for vessel density calculation (please refer to Supplementary Fig. S4A for micrographs). Statistical significance was assessed using *t*-test with Welch’s correction: ****p < 0.0001. Data are representative of three independent experiments. **C** PIM inhibition decreases RS-M’s capacity to endocytose extracellular collagen. THP1-derived M0 macrophages and RS-conditioned macrophages (RS-M) treated as described in (**A**) were incubated with 5 µg/ml Oregon-488- gelatin conjugate for 45 min at 37 °C or on ice. Bars indicate MFIs of 37 °C sample normalized to the MFI of the corresponding sample kept on ice. Error bars indicate the standard deviations of two independent replicates in a representative experiment. *P*-values were determined using the two-sided *t*-test: **p < 0.01; ***p < 0.001. Data are representative of three independent experiments. **D** Pan-PIM inhibitors attenuate RS-M chemoattraction of eosinophils. THP1-derived M0 macrophages and RS-M treated with DMSO or PIM inhibitors were seeded in equal numbers. Next, CFSE-labeled eosinophils (CD16^−^/CD66b^+^) were placed onto 5 µm-pore transwell inserts mounted over macrophages, as shown in a diagram on the left. Following 16 h of incubation, the numbers of migrated CFSE^+^ cells in the bottom chamber were assessed by flow cytometry. Error bars represent ±SD of two independent replicates in a representative experiment. *P*-values were determined using the two-sided *t*-test: **p < 0.01. Data are representative of three independent experiments.

Since PIM inhibition in RS-M downregulated gene expression and protein levels of molecules involved in tumor vessel formation (e.g. IL8, MMP9, CHI3L1/2) [33–36], extracellular matrix remodeling (TIMP1, CD206/mannose receptor C-type 1, MRC1) [16] and eosinophil chemotaxis (IL8, CCL17, CCL24/eotaxin-2, CCL28) [37–41] (Fig. 4C, F), we investigated effects of PIM blockade in RS-M on their ability to support vessel formation, collagen uptake and eosinophil migration. RS-M-conditioned medium induced HUVECs tubulogenesis and this effect was attenuated when the medium was derived from RS-M previously exposed to MEN or PIM447 (Fig. 5A). Consistent with this, CD34^+^ endothelial cells were significantly less abundant in tumors isolated from MEN-treated mice as determined by IHC (Fig. 5B, Supplementary Fig. S4A). As expected, compared to M0 control macrophages, RS-M exhibited increased internalization of extracellular collagen and stimulated eosinophil migration. However, when RS-Ms were pretreated with MEN or PIM447, these activities were markedly decreased (Fig. 5C, D, Supplementary Fig. S4B).

### RS-conditioned macrophages foster Treg differentiation in PIM-kinase-dependent manner

**Fig. 6:**
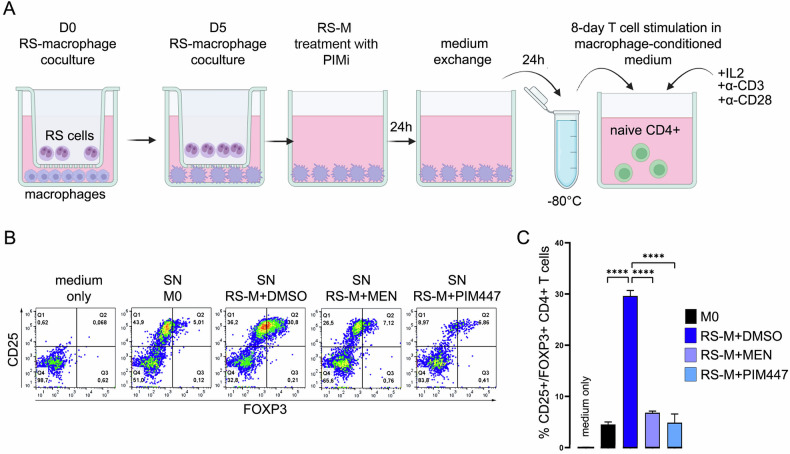
PIM kinases are involved in TAM-mediated differentiation of regulatory T cells. **A** Diagram of the in vitro Treg differentiation assay. M0 macrophages and and RS-conditioned MdMs treated with DMSO or PIM inhibitors for 24 h (MEN – 3 µmol/L, PIM447 – 7 µmol/L) were harvested, washed and seeded in equal numbers in a fresh, inhibitor-free medium for 24 h. Autologous CD4^+^ T cells were stimulated with soluble anti-CD3 and anti-CD28 antibodies in macrophage-conditioned media supplemented with IL2 for 8 days. **B**, **C** PIM inhibitors attenuate the capacity of TAMs to support the development of Tregs. Following 8-day incubation in macrophage-conditioned media, the number of Tregs (CD25^+^/FOXP3^+^/CD4^+^ T cells) was determined by flow cytometry. **C** Bars represent the relative abundance of CD25^+^/FOXP3^+^ cells (% of total CD4^+^ T-cells) in two independent replicates (±SD) in a representative experiment. *P*-values were determined using the two-sided *t*-test: ****p < 0.0001. Data are representative of three independent experiments.

Previous studies demonstrated an increased abundance of functional Tregs in cHL infiltrates [15, 42]. Given the increased expression of TGFβ, IDO1, IL4I1 and CCL17/TARC in primary and in vitro polarized TAMs, we hypothesized that cHL-associated macrophages facilitate induction of Tregs. To test this hypothesis, we adapted an in vitro Treg differentiation assay [13] and evaluated the formation of CD25^+^/FOXP3^+^ cells from CD4^+^ T cells cultured in RS-M-conditioned media by flow cytometry (Fig. 6A). Compared to the control M0-conditioned medium, RS-M-conditioned supernatants increased the percentage of CD25^+^/FOXP3^+^ T cells (25.79% RS-M vs 5.01% in M0 supernatants). In marked contrast, CD4^+^ T cells failed to develop the Treg phenotype when cultured in supernatants conditioned by RS-M that were previously pretreated with MEN or PIM477 (Fig. 6B, C). Consistent with the role of TAMs in Treg induction, number of FOXP3-positive cells was markedly higher in CD68^+^ macrophage-rich cHL biopsies, compared to tumors with lower macrophage numbers (Supplementary Fig. S5). These results indicate that TAMs support Treg formation in cHL and this macrophage-mediated process can be blocked by pharmacological PIM inhibition.

## Discussion

Several recent studies on the cHL TME positioned TAMs at the center of cHL immune evasion [12, 14, 15]. However, TAMs are capable of supporting tumor development in a variety of additional mechanisms, including delivery of antiapoptotic stimuli, fueling tumor cell proliferation, chemoattracting additional TME components, and favoring neovascularization and invasion [43]. In this study, we provide new insights into the features and functions of macrophages in the cHL TME and their relationship with RS cells. We show that paracrine interactions of macrophages with RS cells induce the expression of PIM kinases. PIMs partake in macrophage programming to pro-tumoral TAMs. These RS-educated macrophages exhibit multiple functions supporting tumor growth and express immunomodulatory proteins involved in the development of an immunosuppressive milieu. Importantly, PIM inhibition decreases TAM ability to stimulate angiogenesis, induce eosinophil chemotaxis, Treg development and ECM remodeling.

Although TAMs have been recognized as crucial TME constituents associated with immune evasion and inferior prognosis [11], studies of cHL TAMs have been limited to static immunofluorescence and gene expression profiling. This limitation has its source in the fragility of primary TAMs [42], inability to culture them in vitro, lack of appropriate cHL animal models, plasticity, and heterogeneity of primary TAMs [43]. In addition, primary TAMs resemble transcriptionally other myeloid-derived cells, such as DCs and monocytes, suggesting a continuity of myeloid cell states in cHL [14]. These circumstances prompted us to develop an in vitro model of TAMs, allowing for dynamic studies of their functional, phenotypic and transcriptomic changes. We have shown that our in vitro developed TAM model is a close approximation of primary TAMs, with overlapping gene expression signatures and immunophenotype, including high CD68, CD86, CD206, PD-L1 and IDO1. It should be underscored, however, that the cHL TAM model has certain limitations. Most importantly, primary TAMs are known for their striking heterogeneity, a feature that was not evaluated in studies of the model performed herein. Multiple macrophage transcriptional states were identified in the cHL TME, including a population of inflamed, IL1β^+^ macrophages/monocytes residing near RS cells [22]. Importantly, presence of the precursor monocyte population (termed Cluster 0) with similar transcriptional features was associated with immune checkpoint blockade failure [22]. Although we did not perform single-cell-level analyses of in vitro RS-conditioned TAMs, the obtained cells exhibited marked enrichment of the Cluster 0 macrophage/monocyte signature genes, indicating that at least a part of these cells resemble this pathogenic population.

Second, the gene expression signature used to measure the similarity between in vitro developed TAMs and primary cells is an averaged signature of a myeloid cell cluster, incorporating also DCs and monocytes, indicating that the in vitro developed TAMs exhibit certain features of these cells. However, given the ontological dependencies between monocytes, DCs and macrophages, such similarity is not surprising [44]. Despite these limitations, the model represents a justifiable approximation of primary TAMs, recapitulating their key features, including phenotypic plasticity and swift responses to environmental cues. Thus, the model is a valuable tool for dynamic studies of TAM function. In addition, the construction of the model points to the paracrine TAM programming and the pivotal role of RS cells in this process, although the detailed mechanisms and mediators remain to be elucidated.

Several lines of evidence indicate that TAMs impinge on the efficacy of targeted immunotherapies in cHL through expression of PD-1 and CTLA4 ligands PD-L1/2 and CD86, or IDO1 [12, 13, 15, 22]. Our comprehensive TAM analyses revealed elevated expression levels of additional molecules implicated in the regulation of T-cell immunity, including CD209 and IL4I1 [32, 45–47]. CD209 (DC-SIGN)-expressing macrophages inhibit CD8 T cells immunity and promote Treg cells expansion, fostering allograft tolerance [48]. In invasive bladder cancer, elimination of these DC-SIGN-expressing macrophages reactivated antitumor immunity and improved the efficacy of immunotherapy [47]. IL4I1 is a metabolic immune checkpoint that activates the aryl hydrocarbon receptor (AHR) signaling by the generation of tryptophan catabolites, indole metabolites and kynurenic acid. As AHR promotes Treg differentiation, IL4I1 expression in TME suppresses adaptive immunity [49]. Abundant IDO1 and IL4I1 expression has been reported in cHL TME and a high kynurenine/tryptophan ratio in cHL patients was shown to be associated with their worse progression-free survival [13, 50, 51]. Since an increased abundance of functional Tregs is a hallmark of cHL [13, 42, 52], IDO and IL4I1-expressing macrophages might contribute to the suppression of adaptive immunity in cHL via Tregs induction. Interestingly, expression of IL4I1 is induced by PD-1 blockade, suggesting that it might represent a mechanism of resistance to immune checkpoint inhibitors [49].

In addition to these checkpoint molecules, RS-M express multiple chemokines and cytokines (e.g., CCL2, CCL13, CCL17, CCL24, IL8, CHI3L1/2, MMPs: −9, −19 and −25) mediating chemotaxis of eosinophils, Tregs and other TME constituents, ECM remodeling and vessel formation in cHL. Tissue eosinophilia is a well-established adverse prognostic factor in cH [53]. Mechanistically, eosinophils are a source of TNF superfamily ligands (e.g., CD30L, CD40L), conferring survival and proliferation signals to RS cells [54]. Thus, TAM-induced eosinophil infiltration represents a likely mechanism fostering cHL growth. Finally, RS-M facilitate angiogenesis and matrix remodeling through collagen uptake, likely promoting tumor growth, invasion and disease dissemination via lymphatic vessels [16].

We found that RS-cell-mediated induction of PIM kinases orchestrates the acquisition of these tumor-promoting functions in TAMs. We previously demonstrated that PIM kinases are important signaling hubs in RS cells. PIM pharmacological inhibition downregulated expression/activity of factors involved in survival, immune privilege and interactions of the malignant cells with TME, including macrophage chemoattractants and M2-macrophage-polarizing factors - IL13, CCL5 and CSF1 [18]. This is also in agreement with the effect exerted by MEN/dapolsertib in AML blasts [55]. Consistent with this, PIM inhibition decreased the numbers of monocytes/macrophages in cHL xenografts in vivo and profoundly affected the polarization of macrophages cocultured with RS cells.

Mechanistically, inhibition of PIM kinases in RS-conditioned TAMs decreased the activity of STAT3,6 and CREB1, transcription factors involved in the development of M2-like macrophages [23, 25]. As a consequence of their impaired polarization, these PIM PROTAC or inhibitor-treated macrophages failed to induce the expression of surface and secreted proteins associated with pro-tumoral TAMs, including PD-L1, CD209, CD206, IL6, IL8 or MMP9 [12, 16, 47, 56–58]. Thus, our observations provide further evidence supporting the role of PIMs in orchestrating RS-macrophage crosstalk in cHL TME. In line with these observations, pharmacological PIM blockade in RS-M attenuated stimulation of blood vessel formation, collagen uptake, eosinophil chemotaxis and Treg development. These results indicate that PIM inhibition represents a rational therapeutic strategy to target TAM polarization programs supporting survival, growth, dissemination and immune escape in cHL.

The major limitation of this study is the lack of in vivo evidence that would confirm the reactivation of antitumor immunity in PIM inhibitor-treated animals, either in single-agent setting or in combination with immune checkpoint inhibitors (ICIs). Since syngeneic and patient-derived xenograft (PDX) mouse models for cHL are not available, it is not feasible to address this issue in a disease-specific manner. However, models of other malignancies with similar immunosuppressive features might be useful to address this point. Recently, combination of PIM inhibitor with ICI has been shown to synergistically reduce tumor growth in syngeneic prostate cancer murine model [59]. In this study, macrophage-specific PIM knockout attenuated tumor growth in immunocompetent mice. Furthermore, Chatterjee and coworkers demonstrated that the combination of a PIM kinase inhibitor with an anti-PD1 antibody improves adoptive T-cell therapy and confers long-term tumor control in B16 melanoma model [46]. Interestingly, the observed effects of the combination were attributed to PIM kinase-inhibition-mediated induction of central-memory phenotype in T cells with a concomitant decrease in PD-1 expression. PIM inhibition did not hamper T cell activation but increased their persistence [46]. These findings might be relevant for cHL therapy as memory T cells appear to be required for long-lasting responses to ICIs [60].

**Fig. 7:**
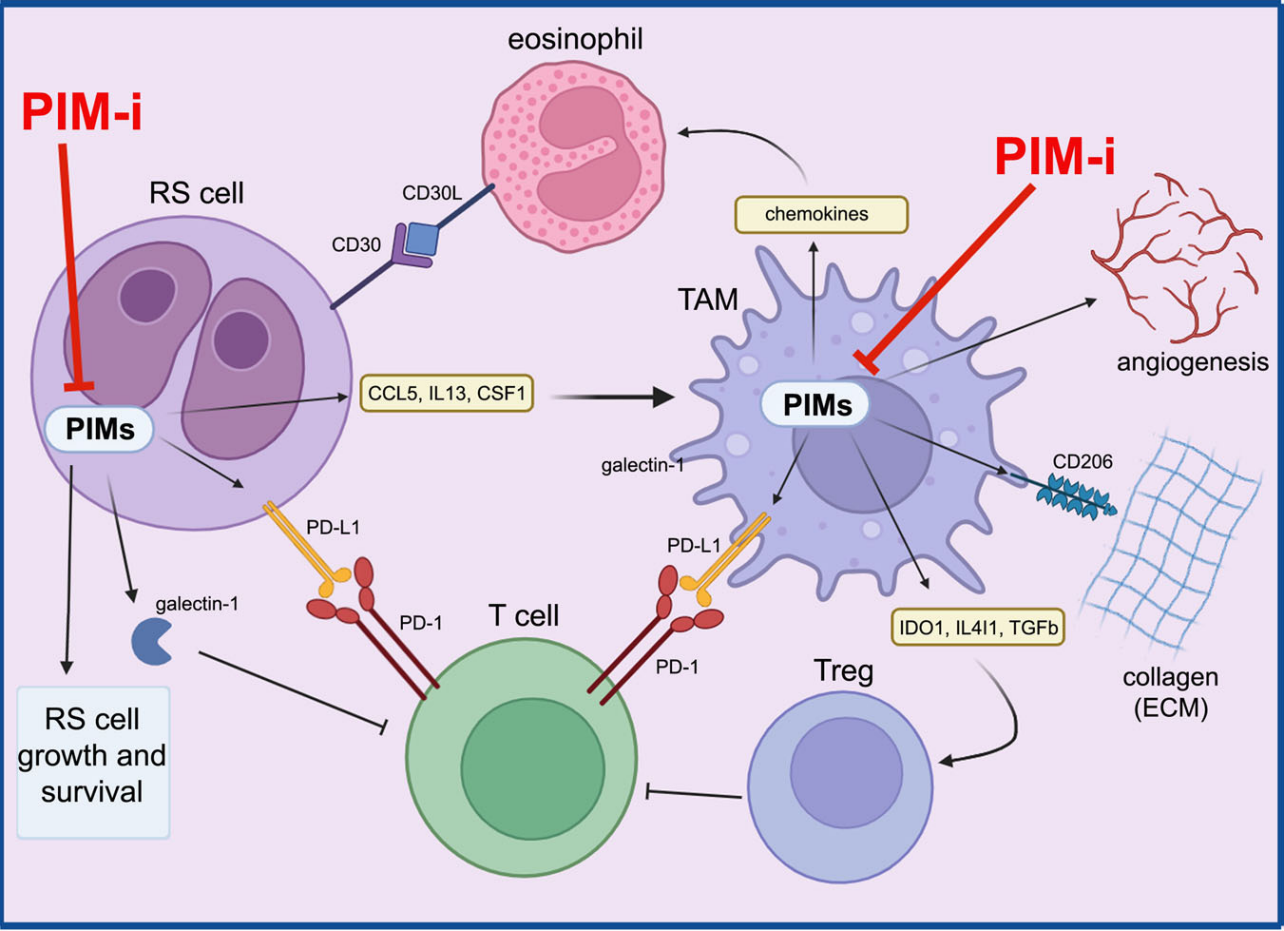
Proposed model of pleiotropic effects of PIM kinase inhibition in cHL. PIM kinases support RS cell growth, survival, immune privilege and are involved in RS-macrophage interactions important for the formation of the pro-tumoral and immunosuppressive features of macrophages. PIM blockade induces RS cell death and impedes TAM-dependent mechanisms contributing to tumor growth and immune escape. Created with BioRender.

Taken together, our former [18] and present studies demonstrate that PIM blockade: (i) induces RS cell death (ii) downregulates the expression of molecules involved in the development of immunosuppressive TME, (iii) perturbs pro-tumoral TAM phenotype, and (iv) attenuates multiple RS- and TAM-dependent mechanisms counteracting T cell activation/response (Fig. 7). These results indicate that pharmacological PIM inhibition may represent a strategy to improve the efficacy of ICIs and other immunotherapies in cHL.

## Supplementary information

Supplemental information - Materials, methods and figures

Supplemental information - immunoblot raw data

Supplemental information - Table 1

Supplemental information - Table 2

Supplemental information - Table 3

Supplemental information - Table 4

Supplemental information - Table 5

## Data Availability

Data and further details regarding the manuscript are available from the corresponding author upon request. Sequencing data are available through indicated repositories.
