# Supplementary material for: PIM kinase inhibition attenuates pro-tumoral and immunosuppressive functions of macrophages in classic Hodgkin lymphoma
Source: Cell Death Dis. 2025 Dec 26;17(1):136. doi: 10.1038/s41419-025-08402-5 (PMC12847870; doi:10.1038/s41419-025-08402-5)
Supplement: Supplementary file 1 — Supplemental information - Materials, methods and figures [file 41419_2025_8402_MOESM1_ESM.docx]

**Supplemental Information**

**PIM kinases support pro-tumoral and immunosuppressive functions of macrophages in classic Hodgkin Lymphoma**

Maciej Szydłowski^1^, Ewa Kurtz^1,2^, Filip Garbicz^1^, Julia Maroszek^1,2^, Michał Pawlak^1^, Natalia Ochocka^3,4^, Marcin Tabaka^3,4^, Monika Prochorec-Sobieszek^5^, Anna Szumera-Ciećkiewicz^6^, Patryk Górniak^1^, Olga Szymańska-Giemza^5^, Grzegorz Rymkiewicz^7^, Agnieszka Kołkowska-Leśniak^8^, Wojciech Kukwa^9^, Ewa Paszkiewicz-Kozik^10^, Justyna Totoń-Żurańska^11^, Sylwia Radomska^5^, Zofia Pilch^12^, Dominika Nowis^13^, Jakub Golab^12^, Michał Kurlapski^14^, Jan Maciej Zaucha^14^, Alicja Braczko^15^, Marcin Kaszkowiak^1,2,16^, Paweł Wołkow^17^, Katarzyna Wiktorska^18^, John Brognard^19^, Sabina Lichołai^1^, Ewa Lech-Marańda^8^, and Przemysław Juszczyński^1^

^1^Dept. of Experimental Hematology, Institute of Hematology and Transfusion Medicine, Warsaw, Poland

^2^Centre of Postgraduate Medical Education, Doctoral School, Warsaw, Poland;

^3^Computational Genomics Group, International Centre for Translational Eye Research – ICTER, Warsaw, Poland

^4^Institute of Physical Chemistry, Polish Academy of Sciences, Warsaw, 01-224, Poland

^5^ Dept. of Diagnostic Hematology, Institute of Hematology and Transfusion Medicine, Warsaw, Poland

^6^ Biobank, Maria Sklodowska-Curie National Research Institute of Oncology, Warsaw, Poland;

^7^ Flow Cytometry Laboratory, Dept. of Cancer Pathomorphology, Maria Sklodowska-Curie National Research Institute of Oncology, Warsaw, Poland

^8^ Dept. of Hematology, Institute of Hematology and Transfusion Medicine, Warsaw, Poland;

^9^Department of Otolaryngology, Faculty of Dental Medicine, Medical University of Warsaw, Warsaw, Poland

^10^ Dept. of Lymphoid Malignancies, Maria Sklodowska-Curie National Research Institute of Oncology, Warsaw, Poland

^11^Center for Medical Genomics OMICRON, Jagiellonian University Medical College, Cracow, Poland

^12^ Dept. of Immunology, Medical University of Warsaw, Warsaw, Poland

^13^ Laboratory of Experimental Medicine, Medical University of Warsaw, Warsaw, Poland

^14^ Dept. of Hematology and Transplantology, Medical University of Gdansk, Gdansk, Poland;

^15^ Dept. of Biochemistry, Medical University of Gdansk, Gdansk, Poland;

^16^ Broad Institute of Massachusetts Institute of Technology and Harvard, Cambridge, Massachusetts, USA

^17^University of Rzeszów, Medical College, Faculty of Medicine, Institute of Medical Sciences, Division of Laboratory Diagnostics and Clinical Epigenetics

^18^ Dept. of Physics and Biophysics, Institute of Biology, Warsaw University of Life Sciences-SGGW, Warsaw, Poland;

^19^ Laboratory of Cell and Developmental Signaling, National Cancer Institute, NIH, Frederick, MD 21702, USA

**Materials and methods**

**Cell lines, culture conditions and chemicals**. Human cell lines THP1, L428 and L1236 were obtained from DSMZ and maintained in RPMI 1640 medium supplemented with 10% FBS (Sigma-Aldrich, cat. F9665). HUVEC-TERT2 were obtained from ATCC and cultured in EBM2 (Lonza, cat. CC-3156) supplemented with EGM2 SingleQuots Supplements (Lonza, cat.# CC-4176) and 3% FBS on plates coated with 0.2% gelatin type B from bovine skin (Sigma-Aldrich, cat.# G9391). All cell lines have been routinely tested for mycoplasma contamination. The pan-PIM inhibitor MEN1703 was provided by Menarini Ricerche (Pomezia, Italy), PIM447 was purchased from Selleckchem and PIM protac SGI-VHL02 was kindly provided by dr John Brognard (NIH, Frederick, USA). All chemicals were dissolved in DMSO (Sigma-Aldrich). In vehicle-control experiments, the maximal final DMSO concentration was 0.03% (control for 7 µM dose).

**Isolation of monocytes and naïve CD4+ T cells**. Peripheral blood mononuclear cells (PBMCs) of healthy donors were isolated from fresh buffy coats by density‐gradient centrifugation using Histopaque-1077 (Sigma-Aldrich, cat.# 10771). CD14+ monocytes and naïve CD4+ T cells were obtained from PBMCs by magnetic cell separation using CD14 microbeads (Miltenyi Biotec, cat.# 130-050-201) and Naive Pan T Cell Isolation Kit (Miltenyi Biotec ,cat.# 130-097-095) followed by CD4+ T cell isolation using CD4 microbeads (Miltenyi Biotec, cat.# 130-097-048), according to the manufacturer’s instructions.

**Macrophage differentiation and generation of *in vitro* cHL-TAMs**. THP1 cells were differentiated into macrophages (THP1-M) using a protocol adapted from a previous study(1). Briefly, THP1 cells were stimulated with 250 nM Phorbol-12-myristate-13-acetate (PMA, Sigma-Aldrich) in RPMI medium for three days, followed by four days resting in PMA-free RPMI medium. To generate normal monocyte-derived macrophages (MdM), CD14+ monocytes from healthy blood donors were incubated with 10 ng/ml CSF-1 (Biolegend) in TexMACS (Miltenyi Biotec) for 7 days. To generate an *in vitro* model of cHL-TAMs, differentiated THP1-M and MdM, expressing a mature macrophage marker 25F9, were cocultured with L428 or L1236 RS cells for five days under conditions precluding direct contacts, using 0.4 µm polycarbonate cell culture inserts (Nunc^TM^, Thermo Fisher Scientific, cat. 140640). For unstimulated, control (M0) macrophages, THP1-M and MdM were cultured in RPMI only.

**Immunoblotting**. Protein lysates were resolved by SDS-PAGE, transferred to PVDF membranes (Millipore) and immunoblotted with primary and appropriate HRP-labelled secondary antibodies (**Supplemental Table 4**). Signals were developed by enhanced luminescence using ECL reagent (Perkin Elmer) and a digital image acquisition system (G:Box, Syngene). To re-probe with another antibody, blots were incubated in the stripping buffer (2% SDS, 62.5 mM Tris/HCl pH=6.8, 0.8% β-mercaptoethanol) at 50°C for 30 minutes and analyzed as described above. Expression of investigated proteins was normalized to GAPDH levels, whereas phosphorylation levels of protein of interest were normalized to expression of the total protein.

**Phospho-protein arrays**. Exploratory analysis of signaling pathway activation in RS-conditioned macrophages was performed using Proteome Profiler Human Phospho-Kinase Array Kit, 500 µg protein lysate per array (A+B) and following original protocol (R&D Systems, cat. ARY003C). Appropriate matched M0 macrophages were used as a reference. Densitometric quantifications of dot intensities were performed using ImageJ software (https://imagej.nih.gov/ij/).

**Flow cytometry**. Prior to the staining with antibodies, macrophages were incubated with FcR blocking reagent according to the manufacturer’s instructions (Miltenyi Biotec, cat.# 130-059-901). Surface Expression of CD163, PD-L1, CD206, CD209 and CD86 on macrophages was assessed as described before(2), using antibodies listed in **Supplemental Table 4** and Cytoflex flow cytometer (Beckman Coulter). For Treg identification, T cells were stained at first with Fixable Yellow live/dead stain (ThermoFisher Scientific, cat.# L34959) and anti-CD25 antibody, followed by intracellular staining using anti-FOXP3 antibody (**Supplemental Table 4**) and Foxp3/Transcription Factor Staining Buffer Set, according to the manufacturer’s instructions (ThermoFisher Scientific, cat.# 00-5523-00). Flow cytometry (FC) data were analyzed using FlowJo software (v.10; TreeStar). Statistical analyses were performed using GraphPad Prism Version 8.

**Cytokine and chemokine detection.** Proteome profiler Human XL cytokine array kit (R&D Systems, cat.# ARY022B) was used to determine the relative expression of multiple cytokines produced by M0 and RS-M. Densitometric quantifications of dot intensities were performed using ImageJ software. For quantitative assessment of IL6, IL8, CCL17, CCL2, MMP9 and TGFβ production in RS-M following incubation with PIM inhibitors, control (M0) and DMSO/PIM inhibitor-treated (24h) macrophages were collected and 0.5×10^5^ cells were suspended in fresh RPMI. After 24h, cell culture supernatants were harvested and levels of secreted factors were determined using bead-based Legendplex assay (Biolegend) and flow cytometry. Capture antibody beads used in the assay are listed in **Supplemental Table 4**. Assay was performed according to the manufacturer’s protocol and flow cytometry data was analyzed using Legendplex Data Analysis Software (Biolegend).

**Metabolic profiling of macrophages**. Seahorse XFp Metabolic Flux Analyzer was used to measure the Oxygen Consumption Rate (OCR) and Extracellular Acidification Rate (ECAR). Macrophages were seeded at a density of 10,000 per well in a final volume of 80 µL on the Seahorse plate one day before the assay. For the mitochondrial stress test, 45 minutes before analysis, growth medium was replaced with Seahorse medium enriched with 1 mM pyruvate, 2 mM glutamine, and 10 mM glucose. After 3 baseline measurements, oligomycin (inhibitor of Complex V), FCCP (mitochondrial uncoupler), and a mix of rotenone and antimycin A (inhibitors of Complexes I and III) were injected sequentially into each well for final concentrations of 1.5 µM, 0.5 µM, and 0.5 µM, respectively. The data obtained at indicated time points were used to calculate ATP-linked respiration, maximal respiration, spare capacity, proton leak, and non-mitochondrial oxygen consumption. For the glycolysis stress test, macrophages were seeded as described above and on the following day, Seahorse XFp RPMI medium was enriched with 2 mM glutamine and adjusted to pH 7.4. Thereafter, glucose (substrate for glycolysis), oligomycin (inhibitor of mitochondrial ATP production), and 2-deoxyglucose (inhibitor of hexokinase) were consecutively added into the wells to final concentrations of 10 mM, 5 µM, and 100 mM, respectively. Obtained data were used to calculate glycolytic capacity, glycolytic reserve, glycolysis, and non-glycolytic acidification. All data were normalized to mg of protein.

***In vitro* tube formation assay.** Control (M0) and RS-conditioned macrophages treated with DMSO or PIM inhibitors for 24h were harvested and seeded at 1×10^5^ in 500 μl EBM2 medium without additives. After 24h, their supernatants were collected and used in a HUVEC tube formation assay. HUVECs were starved for 18h in EBM2 + 0.1% FBS before the assay. Following starvation, cells were detached using trypsin/EDTA (Lonza, CC-5012), centrifuged at 200×g for 5 min, and washed with PBS to remove the remaining trypsin. 5×10^4^ HUVECs were resuspended in 300 μL of EBM2 medium mixed 1:1 with appropriate M0/RS-M conditioned medium. This suspension was then added onto a solid layer of Matrigel Growth Factor Reduced (GFR) Basement Membrane Matrix, phenol red-free and LDEV-free (Corning, cat. #356231), in 24-well plates. At the time of the peak tube formation (6-8 h), the wells were gently washed with PBS and captured at 10× magnification using a Zeiss Primovert microscope equipped with an Axiocam. Tube formation was assessed using the Angiogenesis Analyzer ImageJ plugin.

**Collagen uptake by macrophages.** Assessment of the extracellular collagen uptake by macrophages was performed essentially as described earlier(3). Briefly, M0 and RS-M treated with DMSO or PIM inhibitors for 24h were harvested using trypsin/EDTA, washed twice with PBS and suspended at 1×10^5^ cells/ml in RPMI medium supplemented with Gelatin-Oregon Green 488 conjugate (Sigma-Aldrich, cat.# G13186) at 5 µg/ml. Macrophages were then incubated for 45 min at 37°C/5% CO_2_ or on ice (to block internalization). Following incubation, cells were centrifuged, fixed for 10 min at 37°using Cytofix buffer (BD, cat.# 554655), washed twice with PBS and subjected to FC analysis.

**Eosinophil migration**. Human eosinophils were isolated from healthy donor-derived PBMCs using EasySep Human Eosinophil Isolation Kit (Stemcell Technologies, cat.# 17956), according to the manufacturer’s protocol. Purified eosinophils (≥80% CD16-/CD66b+ cells as determined by flow cytometry) were CFSE-labelled as described(4) and suspended in RPMI medium. M0 and RS-M treated with DMSO or PIM inhibitor for 24h were harvested, washed twice with PBS and suspended at the density of 1×10^5^ cells in 1.5 ml RPMI per well of 6-well plate. After 6 hours, 5 µm pore transwell inserts (Sarstedt, cat.# 3930500) were mounted over wells and 1×10^4^ eosinophils in 1 ml RPMI were seeded per insert. After 16 hours of incubation, the numbers of CSFE+ eosinophils in the supernatant of the macrophage compartment were determined by flow cytometry under constant flow rates and in 30s intervals.

**Treg differentiation**. M0 macrophages and MdMs treated with DMSO or PIM inhibitor for 24h were harvested using trypsin/EDTA, washed twice with PBS and suspended at 2×10^5^ cells/ml in supplemented RPMI. After 24 hours, macrophage-conditioned supernatants were collected and stored in aliquots at -80°C. Autologous CD4+ T cells were incubated in either RPMI supplemented with 10% FBS and IL-2 (100 ng/ml, Peprotech, cat.# 200-02) or in macrophage supernatants diluted 1:1 with supplemented RPMI, in the presence of soluble monoclonal antibodies to CD3 (1 µg/ml, UCHT1, BioLegend, cat.# 300402) and CD28 (1 µg/ml, CD28.2, BioLegend, cat.# 302902). IL-2 was added every three days. At the end of day 8, T-cells were washed and analyzed by flow cytometry.

**RNA-seq analysis, GSEA and hypergeometric enrichment analysis**. RNA was isolated using a GeneMATRIX Universal RNA purification kit (EURx). RNA integrity was assessed using TapeStation 4200 Instrument (Agilent). Samples with minimal RIN^e^ value ≥9.7 were used for library preparation. Libraries were generated using SENSE mRNA-Seq Library Prep Kit (Lexogen, cat.# 001.96) and sequenced using NextSeq™ 500/550 High Output Kit v2.5 chemistry (Illumina, cat.# 20024907) on Illumina NextSeq 500 System. RNA-seq reads were aligned to GRCh37 human genome version and gene counts were generated using STAR under nextflow nf-core RNA-seq analysis pipeline(5). Gene count normalization and differential gene expression analysis were performed using DESeq2 and transcripts with FDR-adjusted p value <0.05 were considered significant(6). Volcano plots were generated using ggplot2, heatmaps using pheatmap or heatmap.2 (CRAN R repository). RNA-seq reads are stored in Gene Expression Omnibus (GEO) repository under GSE288623 accession number.

GSEA was performed with ClusterProfiler and fgsea packages as described(7–9). Briefly, interrogated transcriptional signatures were pre-ranked and ordered by gene expression log2 fold change between given experimental conditions (as determined by DESeq2). The pre-ranked signature was then used to calculate whether a priori defined sets of genes (derived from MsigDB, <https://www.gsea-msigdb.org/gsea/msigdb>) or publications indicated in the text) show statistically significant enrichment at either end of the ranking. Hypergeometric enrichments were calculated using hypeR package(10). R codes are available upon request or through github page.

**CITE-seq analysis**.

**Sample preparation and sequencing**. For single-cell transcriptome and epitope tag sequencing, lymph node biopsies from 9 patients with histologically confirmed, newly diagnosed classic Hodgkin Lymphoma (**Supplemental Table 1**) and 3 reactive tonsils from patients who underwent tonsillectomy were included in this study. Tissues were mechanically dissociated and cryopreserved as single-cell suspensions, as described previously(11). Institutional review board approval was obtained for the collection and analysis of tissue samples (Decision number 22/2019). Cell suspensions from cHL tumors and tonsils were rapidly thawed at 37°C, washed in prewarmed RPMI supplemented with FBS (1:1 volume) and dead cells were eliminated using a dead cell removal kit (Miltenyi Biotec, cat.# 130-090-101. Following live cell isolation (≥90% viability confirmed via trypan blue staining), cells were centrifuged 10 min/150×g at 4°C, suspended in 45 µl cell staining buffer (BioLegend cat.# 420201) and incubated for 10 min at 4°C with 5 µl Human TruStain FcX™ Fc Blocking reagent (BioLegend, cat.# 422301). Next, cells were centrifuged as above and suspended in 48 µl cell staining buffer. Each cHL sample and pooled reactive tonsil (control) sample (generated by mixing cells from 3 donors in 1:1:1 ratio) was incubated with unique hashtag antibodies (1 µg/1×10^6^ cells) for 30 min at 4°C. After staining, cells were washed twice, suspended in 1 ml cell staining buffer and combined in equal cell ratios (three cHL samples plus one control tonsil sample per reaction in subsequent GEM generation). Following additional wash, multiplexed samples (5×10^5^ cells) were stained with TotalSeq^TM^-B Human Universal Cocktail, V1.0 according to manufacturer’s recommendations (BioLegend, cat.# 399904). After staining, cells were washed 3 times and suspended in 100 µl PBS/0.04%BSA. Samples with cell viability above 90% were subjected to GEM generation using Chromium X controller (10X Genomics), followed by construction of gene-expression and cell-surface libraries, according to Chromium Next GEM Single Cell 3’ protocol with Feature Barcode technology for Cell Surface Protein (10X genomics, CG000317 Rev.A). Three separate gene-expression and the corresponding cell-surface libraries, each including 3 individual cHL samples and control pooled tonsil sample were sequenced using NovaSeq Sequencing System and S1 Reagent Kit v1.5 (Illumina, cat.# 20028319).

**Data processing and analysis**. Raw sequencing data (BCL files) were demultiplexed into FASTQ with the mkfastq function (CellRanger v7.2.0). The sequencing reads were mapped to a human genome GRCh38 (refdata-gex-GRCh38-2020-A, 10X Genomics) and processed with a CellRanger v.7.2.0 to generate count matrices. Further analysis was performed in R v4.2.2 with Seurat v4.4.0. Filtered count matrices were split into RNA, antibody-derived tag (ADT), and hash-tag oligonucleotide (HTO) data. The HTO data were normalized with the CLR method, and the normalized HTO reads were demultiplexed with HTODemux. Based on the demultiplexing results, sample identities were assigned to each cell based on the HTO indices, whereas multiplets and empty droplets were removed. Poor quality cells with mitochondrial gene content >10% and the number of transcripts (nFeatures) <200 or >6000 were filtered out. ScRNA-Seq data were normalized with the LogNormalization and ADT data with the CLR method. Next, data generated in this study were integrated with reference human tonsils scRNA-seq dataset (GSE165860) by computing the integration anchors with ‘FindIntegrationAnchors’ and integrating the matrices with ‘IntegrateData’ function (Seurat v4.4.0)(12). Dimensionality reduction was performed using the PCA algorithm on the scRNA-seq data. The first 30 principal components were selected based on the elbow plot and used for downstream analysis. Clustering was conducted using the Leiden algorithm at a resolution of 0.7. For visualization, UMAP was applied to the PCA-reduced data to create 2D plots. Cell type annotation was executed through a three-step process: First, we applied the CellTypist v1.6.2 automatic cell type annotation algorithm(13). Second, cell type annotations were transferred from the reference human tonsil dataset. Third, the cell type labels were verified by examining the top differentially expressed genes and highly expressed proteins in each cluster. For tumor and healthy control comparison, differential gene expression analysis was performed with the ‘FindMarkers’ (Seurat v.4.4.0) function using the ‘MAST’ package and min.pct threshold of 0.2.

**Animal studies**. For *in vivo* assessment of MEN1703 effects on monocyte/macrophage tumor infiltration and angiogenesis, 5×10^6^ of L428 cells in a 30% Matrigel Matrix (Corning, cat.# 354230) were inoculated subcutaneously into NSG mice. Five days after inoculation, animals with established disease (tumor volume ≥100mm^3^, n=12) were divided into 2 cohorts with equal average tumor sizes and administered 75 mg/kg MEN1703 or vehicle (H_2_O) for 21 days, via oral gavage using a flexible cannula (Instech Laboratories, Inc.) After the last dose, mice were euthanized and tumor tissues were harvested. Part of the tumor tissue was formalin-fixed for immunohistochemical (IHC) analyses, and another portion was placed in cold PBS, minced with scissors, and subjected to single-cell separation followed by flow cytometry (FC) analysis. For single-cell preparation, explanted tumor tissues were digested and dissociated in DMEM supplemented with collagenase (400U, Sigma-Aldrich, cat.# C5132) and DNAseI (600U, Sigma-Aldrich, cat.# DN25) using GentleMACS instrument (Miltenyi Biotec, program: m_intestine_01, 37°C for 40 min). After dissociation, cell suspensions were filtered through a 70µm cell strainer (BD). Cells were first stained with Zombie NIR™ (BioLegend, cat.# 423105) for viability assessment, then incubated with blocking buffer (PBS/5% rat serum) for 20 minutes. After blocking, cells were stained with antibody cocktail: CD45, F4/80, CD11b, Ly6G, Ly6C and MHC-II (**Supplemental Table 4**). Stained cells were analyzed by flow cytometry using a BD LSRFortessa™ X-20 cell analyzer. Animal studies were performed according to the protocol approved by the local bioethical committee. Animals were housed in controlled environmental conditions, in specific-pathogen-free (SPF) animal facility of the Medical University of Warsaw with water and food provided ad libitum. The experiments were performed in accordance with the guidelines approved by the 2nd Local Ethics Committee in Warsaw and in accordance with the requirements of the EU (Directive 2010/63/EU) and Polish (Dz. U. poz. 266/15.01.2015) legislation.

**Immunohistochemistry and fluorescence microscopy**. Immunohistochemistry (IHC) of formalin-fixed, paraffin-embedded tumor sections explanted from mice was performed as described(4). All microphotographs were taken by a microscope DP73 Olympus BX53 camera (Olympus, Japan). Blood vessels were detected using anti-CD34 antibody (Abcam, cat.# ab81289) and tumor vessel density was assessed using Fiji (ImageJ) software and vessel analysis plug-in (<https://imagej.net/plugins/vessel-analysis>).

CHL tissue microarrays (TMAs) were used for the detection of PIM kinases in macrophages. TMAs were generated from archival formalin-fixed paraffin-embedded (FFPE) biopsy specimens from newly diagnosed, untreated cHL tumors and were reviewed independently by 2 board-certified hematopathologists (M.P.S. and A.S.C.). Following deparaffinization and antigen retrieval, slides were incubated in blocking buffer (PBS/1% BSA/2% FBS/0.1% Triton X-100) for 1h. After washing in PBS/0.05% Tween20 (3×15 min) slides were stained overnight with primary antibodies: PIM1-AF488 (Santa Cruz, cat.# sc-374116), PIM2 (Cell Signalling, cat.# 4730), PIM3 (Cell Signalling, cat.# 4165) and CD68-AF594 (Santa Cruz, cat.# sc-20060). For PIM2 and PIM3 detection, after staining with primary antibodies, slides were washed (3×15 min in PBS/0.05% Tween20) and incubated with VectaFluor^TM^ Excel Amplifier DyLight^TM^ 488, according to the manufacturer’s instructions (Vector laboratories, cat.# DK-1488). Finally, slides were washed twice with PBS, stained for 10 min with Propidium Iodide (0.5 µg/ml in PBS), washed twice with PBS and covered with mounting medium (90% glycerol in PBS). Image acquisition was performed using a Leica TCS SP2 confocal microscope, equipped with 100× oil immersion objective. The fluorescence signal was collected in sequential mode, with the bandpass filter adjusted to the emission spectra of chromophores to avoid spectral spill-over. At least 10 scans of the specimen were performed and the most representative was recorded in line averaging mode to increase the signal-to-noise ratio of the final image.

IHC analysis of FOXP3 and CD68 expression was performed on the cHL cohort (whole tissue sections) reported previously(2), using the following antibodies: anti-FOXP3 ( Abcam, clone 22510) and anti-CD68 (Dako, clone PG-M1) and using an automated stainer (Dako Dennmark A/S). EnVision Detection System (Dako Dako Denmark A/S) was used for signal detection. Stained cHL sections were reviewed independently by 2 hematopathologists (M.P.S. and O.S.G.) and FOXP3 and CD68 expression were determined. Staining controls included human tonsils. Immunohistochemical scoring for CD68: <5% (score 1), 5-50% (score 2). Samples were considered positive for FOXP3 if more than ≥10% of lymphocytes were strongly positive. All microphotographs were taken using a DP72 Olympus BX63 microscope (Olympus, Japan).

**Real-time, quantitative polymerase chain reaction (qPCR).** RNA was extracted using a GeneMATRIX Universal RNA purification kit (EURx) and reverse-transcribed using a Superscript III RT (Invitrogen) kit, according to the manufacturer’s instructions. CSF1, CCL5, IL-13 and YWHAZ (a housekeeping control/internal standard) expression levels were measured in triplicates with the CFX RT-PCR system (BioRad) using the SYBR Green PCR Master Mix (BioRad) and the gene-specific primers (sequences are listed in Supplemental Table 5). Obtained CT values for target genes and the housekeeping control (YWHAZ) were used to calculate relative transcript abundance using 2^-ΔΔCT^ method.

**Supplemental Figure 1**


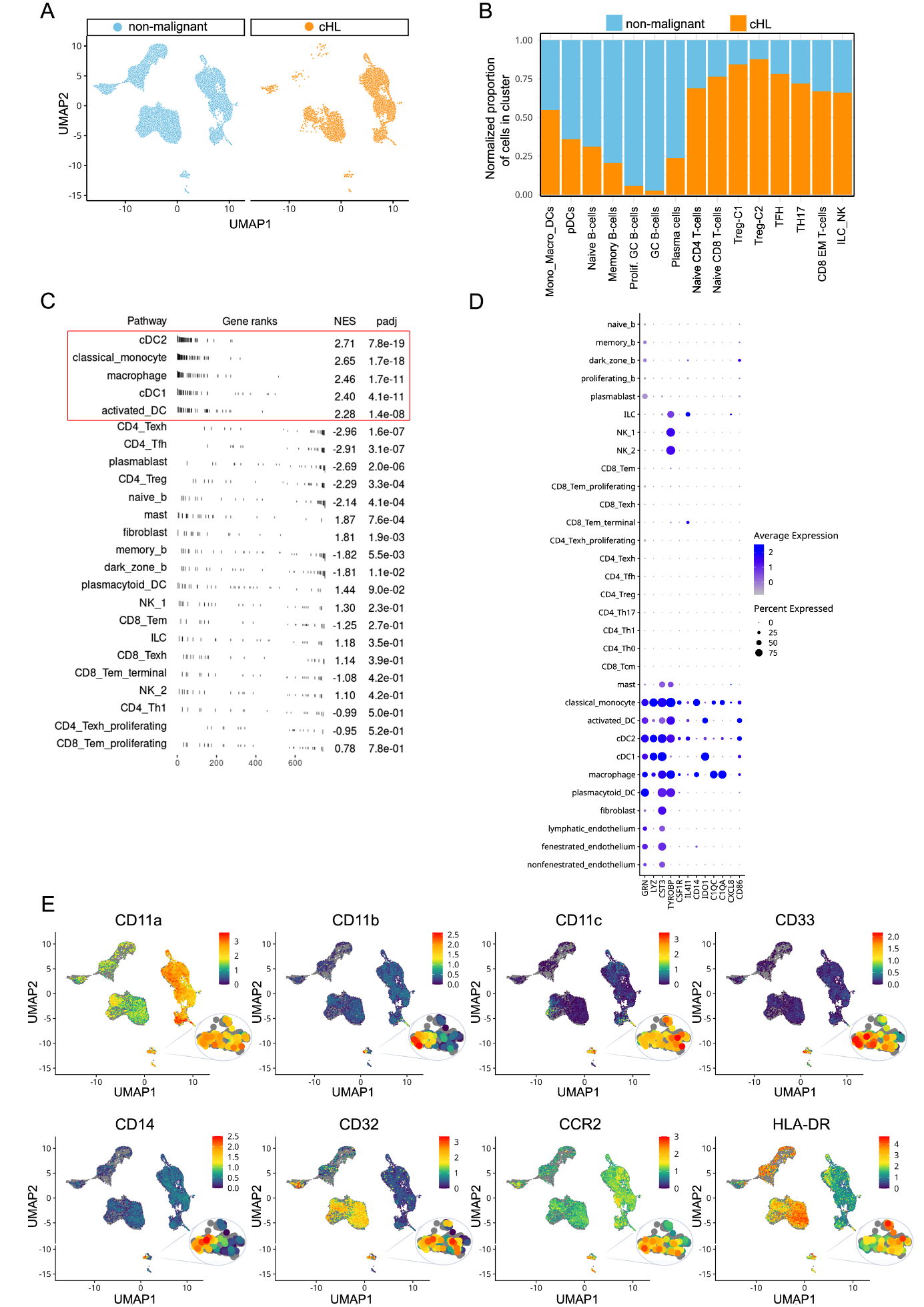


**Supplemental Figure 1**. Identification and characterization of cHL tumor-associated macrophages by CITE-seq.

(A) UMAP plots of all cHL (orange) and RLN (non-malignant, blue) cells. Subsets of cells from cHL and RLN samples are shown on the same coordinates.

(B) Proportion of cells in identified clusters originating from cHL (orange) and RLN (non-malignant, blue) samples.

(C) Enrichment analysis of cHL cell cluster marker genes from Stewart et al ^14^ (top 50 with the highest expression in each cluster) in the M/M/cDC signature. Genes up- and downregulated in M/M/cDC cluster (compared to other clusters) in the CITE-seq dataset (Figure 1) were ranked and ordered by log2-fold change from the genes with highest to the lowest expression (shown in X-axis). Enrichment of Stewart cluster marker genes (shown on Y-axis) in this dataset was evaluated using GSEA. Positions of the genes from MNP subclusters (cDC, classical monocyte, macrophage, cDC1 and activated DC) are significantly skewed toward the left in M/M/cDC cluster (marked with red box), reflecting their selective upregulation in these cells.

(D) Bubble plot showing average expression levels (color intensity) and percent of cells expressing M/M/cDC marker genes (bubble size) in the scRNA-seq dataset from the study by Stewart et al(14).

(E) Expression of macrophage/TAM-associated cell surface proteins. UMAP plots showing antibody-derived signals for the indicated CITE-seq antibodies projected on the plot from Figure 1A. Color scale indicates measured expression levels.

Supplemental Figure 2


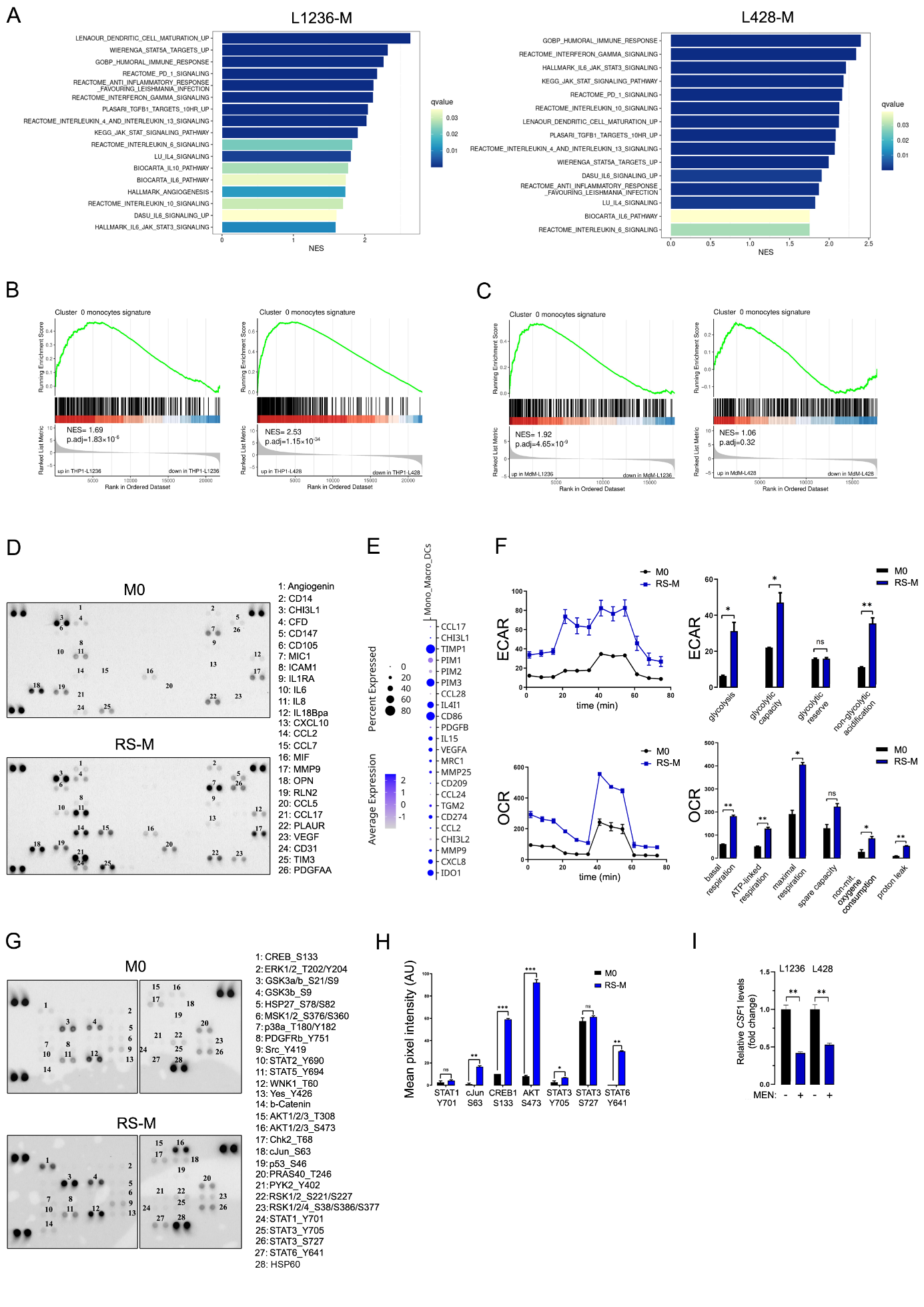


**Supplemental Figure 2**. Characterization of *in vitro* cHL-TAM models and effects of PIM inhibition on macrophage recruitment and programming.

(A) GSEA analyses of signaling pathways upregulated in L1236 and L428-conditioned THP1-derived macrophages. NES: normalized enrichment score.

(B-C) RS-conditioned THP1 and monocyte-derived macrophages overexpress an inflammatory signature typical for pathogenic monocytes and macrophages (Cluster 0 monocytes from Paczkowska *et al*(15)). Genes up and -downregulated control (M0) vs L1236 or L428 – conditioned macrophages were ranked by log2 fold change and used to calculate the enrichment of Cluster 0 signature gene set. The green line indicates the running enrichment score. NES: normalized enrichment; p.adj: BH-corrected p-value.

(D) Raw images from array-based analysis of cytokines and secretory mediators released by L1236-conditioned THP1 derived macrophages, compared to M0 (see Figure 2I for quantification). The signal strength of each spot reflects the expression level of one of the 105 tested secreted factors in the array. Positive and negative spots are located on each corner of the blot. Numbers indicate positions of spots (duplicates) for the given factor.

(E) Bubble plot showing expression of genes associated with TAM functions in primary cells from M/M/DC cluster in CITE -Seq analysis. Average expression levels are indicated by color intensity and the fraction of cells expressing a given gene is indicated by the bubble size. Expression of these genes was also confirmed in *in vitro* developed RS-conditioned THP1 or MdM-derived macrophages (Figure 2H).

(F) Extracellular Acidification rates (ECARs) and oxygen consumption rates (OCRs) of THP1-derived M0 and RS-M. The data obtained at indicated time points were used to calculate glycolytic capacity, glycolytic reserve, glycolysis, non-glycolytic acidification, basal-, ATP-linked- and maximal respiration, spare capacity, proton leak and non-mitochondrial oxygen consumption. Bars to the right represent mean values ±SD of two independent replicates in a representative experiment. P values were determined using the one-way ANOVA: *P<0.05; **P<0.01.

(G) Transcription factors and pathways activated in THP1-derived L1236 conditioned macrophages, compared to M0. Positive and negative spots are located on each corner of the blot. Numbers indicate positions of spots (duplicates) for the given phospho-protein.

(H) Quantification of pixel intensities of the indicated phospho-proteins from array shown in (G). Bars represent mean intensity values ±SD of two replicates (spots), assessed using ImageJ software. P values were determined using the two-sided t-test: *P<0.05; **P<0.01; ***P<0.001.

(H) MEN1703 downregulates expression of *CSF1* in RS cells. L1236 and L428 RS cells were incubated with DMSO (-) or 3 µmol/L MEN1703 (MEN, +) for 24h. Thereafter, expression of *CSF1* was assessed by qPCR. P values were determined using the two-sided t-test: **P<0.01.

Supplemental Figure 3


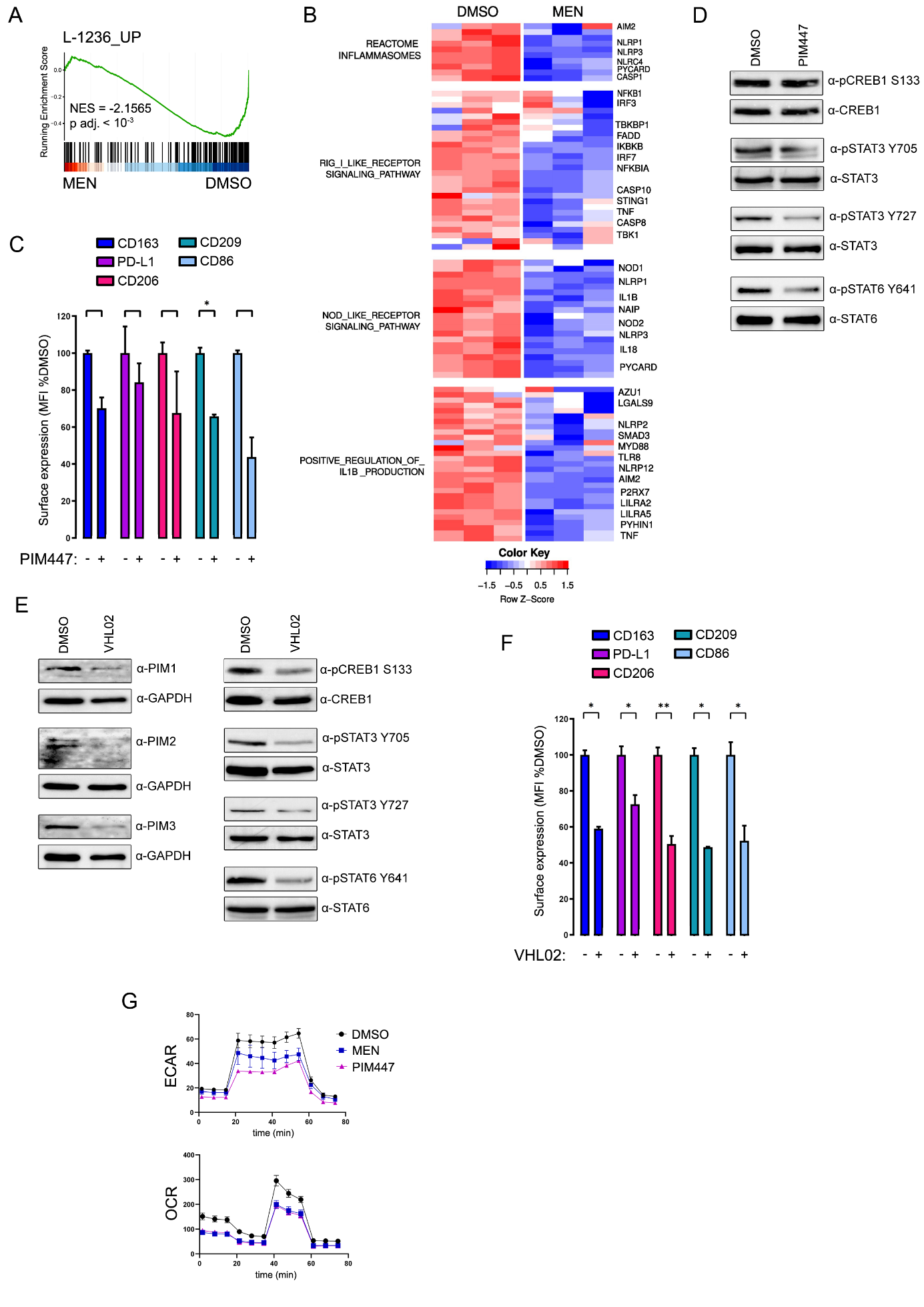


**Supplemental Figure 3**. Pharmacological PIM inhibition or targeted PIM degradation in RS-conditioned macrophages attenuates their pro-tumoral features.

(A) PIM inhibition decreases the expression of RS-M signature genes. L1236-conditioned THP1-derived macrophages were treated with DMSO or 3 µmol/L MEN1703 (MEN) for 24h and subjected to gene expression profiling by RNA seq. Genes up and -downregulated in control (DMSO) vs MEN-treated RS-M were ranked by log2 fold change and used to calculate the enrichment of L1236-MDM signature. The green line indicates the running enrichment score. NES: normalized enrichment; p.adj: BH-corrected p value.

(B) The heat map showing expression of genes associated with innate immune response, NOD- and RIG-I inflammasomes, IL-1β processing/production in L1236-conditioned macrophages following exposure to MEN (as described in A). Color scale indicates relative gene expression levels.

(C) PIM447 modulates RS-M immunophenotype. THP1-derived RS-M were treated with DMSO or 7 µmol/L PIM447 for 24 hours. Surface expression of CD163, PD-L1, CD206, CD209 and CD86 was assessed by flow cytometry. Bar graphs depict MFI of indicated antibodies, normalized to isotype control and relative to DMSO treatment. Error bars indicate ±SD of two independent replicates in a representative experiment. P values were determined using the two-sided t-test: *P<0.05. Data are representative of three independent experiments.

(D) Effects of PIM447 treatment of RS-M on activity of M2 macrophage-associated transcription factors. THP1-derived RS-M were treated with PIM447 as described in (C), harvested and lysed. Phosphorylation levels of CREB (S133), STAT3 (Y705 and S727), STAT6 (Y641) and the corresponding total protein levels were assessed by immunoblotting. Data are representative of three independent experiments.

(E) Effects of targeted PIM kinase degradation in RS-M on the activity of M2 macrophage-associated transcription factors. THP1-derived RS-M were treated with 6 µmol/L PIM PROTAC – SGI-VHL02 for 24 hours, harvested and lysed. The abundance of PIM-1, -2 and -3 kinases, phosphorylation levels of CREB (S133), STAT3 (Y705 and S727), STAT6 (Y641) and the corresponding total protein levels were assessed by immunoblotting. Data are representative of three independent experiments.

(F) PIM degradation decreases the expression of surface proteins induced in macrophages by RS cells. THP1-derived RS-M were treated with DMSO alone or 6 µmol/L SGI-VHL02 for 24 hours. Afterwards, surface expression of CD163, PD-L1, CD206, CD209 and CD86 was assessed by flow cytometry. Bar graphs depict the MFI of indicated antibodies, normalized to isotype control and relative to DMSO treatment. Error bars indicate ±SD of two independent replicates in a representative experiment. P values were determined using the two-sided t test: *P<0.05; **P<0.01. Data are representative of three independent experiments.

(G) Pharmacological PIM inhibition affects the metabolic profile of RS-educated macrophages. THP1-derived RS-M were treated with DMSO, 3 µmol/L MEN or 7 µmol/L PIM447 for 24 hours. Next, cells were harvested and extracellular acidification rate (ECAR) and oxygen consumption rates were assessed using Seahorse XFp metabolic flux analyzer.

Supplemental Figure 4


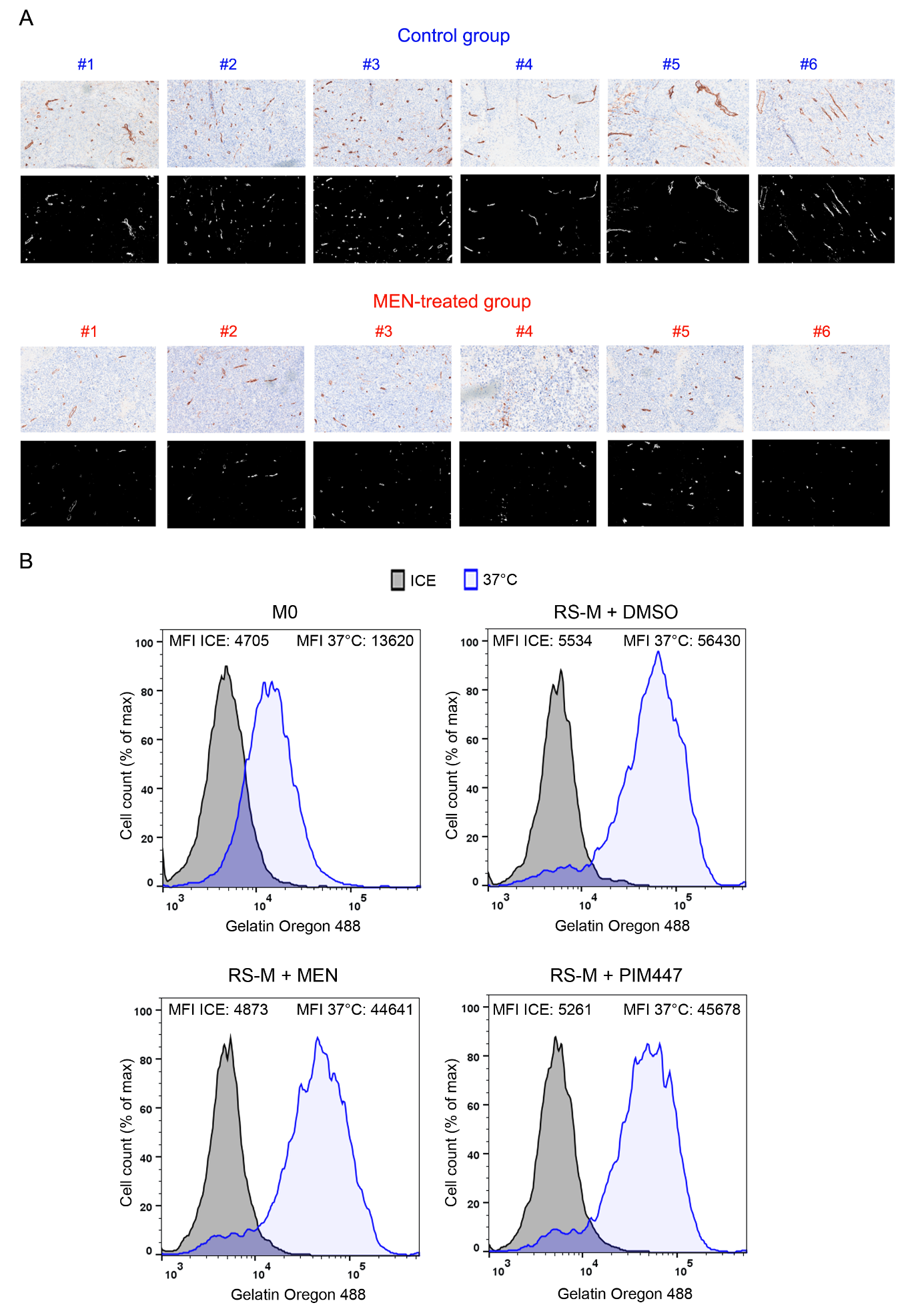


**Supplemental Figure 4**. Effects of the pharmacological PIM inhibition on the formation of blood vessels in cHL tumors and the ability of macrophages to endocytose extracellular collagen.

(A) MEN attenuates tumor vessel formation *in vivo*. NSG mice (n=12) were inoculated with L428 RS cells and treated with MEN (75 mg/kg) or H_2_O (vehicle) for 21 days (n=6 per group). Thereafter, mice were euthanized, tumors were harvested and blood vessels in tumor sections were visualized using anti-CD34 antibody by immunohistochemistry. Original photographs and processed images (below the original) used to calculate blood vessel density are shown. Original magnification was 40×.

(B) PIM inhibition decreases RS-M capacity to endocytose extracellular collagen. MdM M0 and RS-M were treated with DMSO alone, 3 µmol/L MEN or 7 µmol/L PIM447 for 24 hours. Next, macrophages were collected and incubated with 5 µg/ml Oregon-488-labelled gelatin for 45 min at 37°C or on ice. Following incubation, collagen internalization by macrophages was assessed by flow cytometry.

**Supplemental Figure 5**

**Supplemental Figure 5**. Immunohistochemical analysis of FOXP3 and CD68 expression in cHL tumors. Immunohistochemical scoring for CD68: <5% (score 1), 5-50% (score 2). Statistical difference between the groups was assessed using Mann-Whitney-Wilcoxon rank sum test and GraphPad Prism v.8 software.

Supplemental references:

1. Daigneault M, Preston JA, Marriott HM, Whyte MKB, Dockrell DH. The identification of markers of macrophage differentiation in PMA-stimulated THP-1 cells and monocyte-derived macrophages. PLoS One. 2010 Jan 13;5(1):e8668.

2. Szydlowski M, Prochorec-Sobieszek M, Szumera-Cieckiewicz A, Derezinska E, Hoser G, Wasilewska D, et al. Expression of PIM kinases in Reed-Sternberg cells fosters immune privilege and tumor cell survival in Hodgkin lymphoma. Blood. 2017/07/13 edn 2017 Sept 21;130(12):1418–29.

3. Arlt A, von Bonin F, Rehberg T, Perez-Rubio P, Engelmann JC, Limm K, et al. High CD206 levels in Hodgkin lymphoma-educated macrophages are linked to matrix-remodeling and lymphoma dissemination. Mol Oncol. 2020 Mar;14(3):571–89.

4. Szydłowski M, Garbicz F, Jabłońska E, Górniak P, Komar D, Pyrzyńska B, et al. Inhibition of PIM Kinases in DLBCL Targets MYC Transcriptional Program and Augments the Efficacy of Anti-CD20 Antibodies. Cancer Res. 2021 Oct 8;canres.1023.2021.

5. Ewels PA, Peltzer A, Fillinger S, Patel H, Alneberg J, Wilm A, et al. The nf-core framework for community-curated bioinformatics pipelines. Nat Biotechnol. 2020 Mar;38(3):276–8.

6. Love MI, Huber W, Anders S. Moderated estimation of fold change and dispersion for RNA-seq data with DESeq2. Genome Biol. 2014;15(12):550.

7. Piechna K, Żołyniak A, Jabłońska E, Noyszewska-Kania M, Szydłowski M, Żerek B, et al. Activity and rational combinations of a novel, engineered chimeric, TRAIL-based ligand in diffuse large B-cell lymphoma. Front Oncol. 2022;12:1048741.

8. Kim D, Paggi JM, Park C, Bennett C, Salzberg SL. Graph-based genome alignment and genotyping with HISAT2 and HISAT-genotype. Nat Biotechnol. 2019 Aug;37(8):907–15.

9. Yu G, Wang LG, Han Y, He QY. clusterProfiler: an R package for comparing biological themes among gene clusters. OMICS. 2012 May;16(5):284–7.

10. Federico A, Monti S. hypeR: an R package for geneset enrichment workflows. Bioinformatics. 2020 Feb 15;36(4):1307–8.

11. Fromm JR, Kussick SJ, Wood BL. Identification and purification of classical Hodgkin cells from lymph nodes by flow cytometry and flow cytometric cell sorting. Am J Clin Pathol. 2006 Nov;126(5):764–80.

12. Stuart T, Butler A, Hoffman P, Hafemeister C, Papalexi E, Mauck WM, et al. Comprehensive Integration of Single-Cell Data. Cell. 2019 June 13;177(7):1888-1902.e21.

13. Xu C, Prete M, Webb S, Jardine L, Stewart BJ, Hoo R, et al. Automatic cell-type harmonization and integration across Human Cell Atlas datasets. Cell. 2023 Dec 21;186(26):5876-5891.e20.

14. Stewart BJ, Fergie M, Young MD, Jones C, Sachdeva A, Blain A, et al. Spatial and molecular profiling of the mononuclear phagocyte network in classic Hodgkin lymphoma. Blood. 2023 May 11;141(19):2343–58.

15. Paczkowska J, Tang M, Wright KT, Song L, Luu K, Shanmugam V, et al. Cancer-specific innate and adaptive immune rewiring drives resistance to PD-1 blockade in classic Hodgkin lymphoma. Nat Commun. 2024 Dec 30;15(1):10740.
