# Supplementary material for: PIM kinase inhibition attenuates pro-tumoral and immunosuppressive functions of macrophages in classic Hodgkin lymphoma
Source: Cell Death Dis. 2025 Dec 26;17(1):136. doi: 10.1038/s41419-025-08402-5 (PMC12847870; doi:10.1038/s41419-025-08402-5)
Supplement: Supplementary file 2 — Supplemental information - immunoblot raw data [file 41419_2025_8402_MOESM2_ESM.pdf]

Supplemental Information

Immunoblot raw data

Related to Figure 2G

PIM1 (THP1)

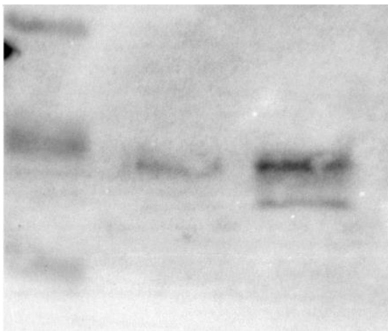

GAPDH for PIM1 (THP1)

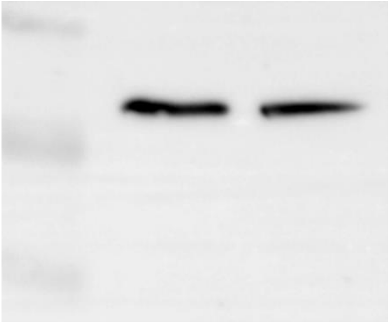

PIM2 (THP1)

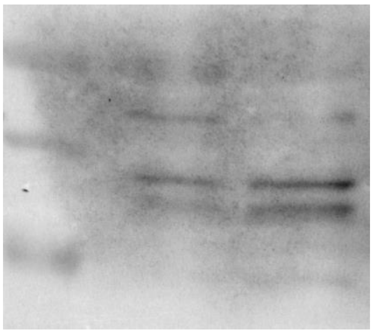

GAPDH for PIM2 (THP1)

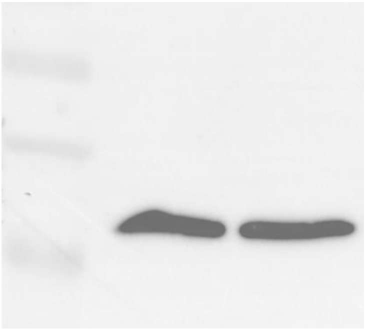

PIM3 (THP1)

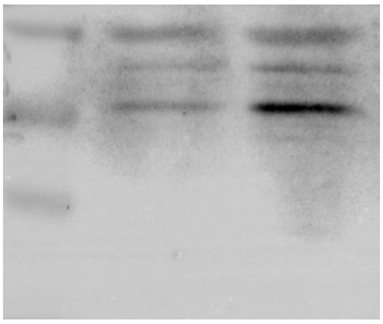

GAPDH for PIM3 (THP1)

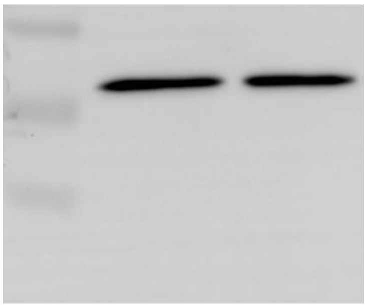

Related to Figure 2G (continued)

PIM1 (MdM)

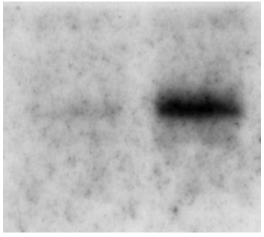

PIM1 (MdM) +ladder

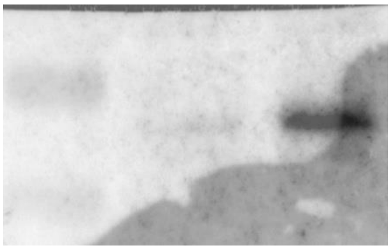

GAPDH for PIM1 (MdM)

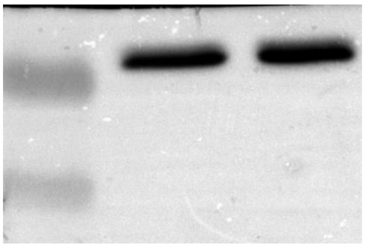

PIM2 (MdM)

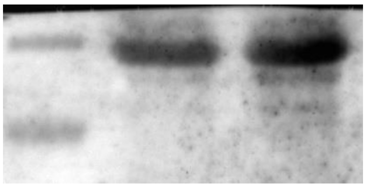

GAPDH for PIM2 (MdM)

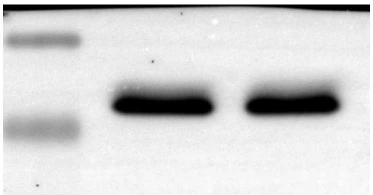

PIM3 (MdM)

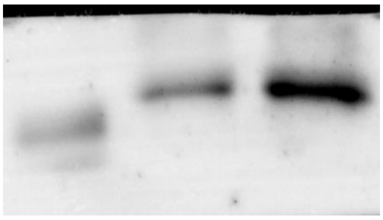

GAPDH for PIM3 (MdM)

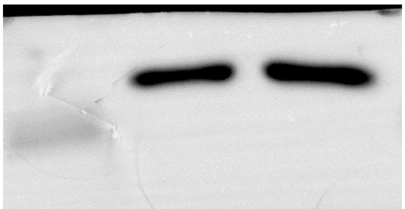

Related to Figure 3A

pCREB1 S133 (MEN)

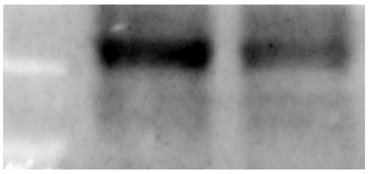

CREB1 (MEN)

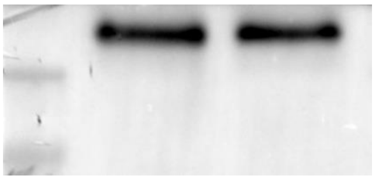

pSTAT3 Y705 (MEN)

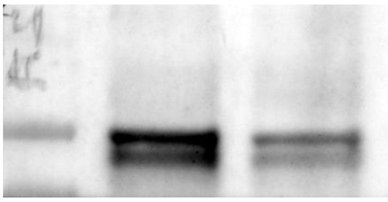

STAT3 for pSTAT3 Y705 (MEN)

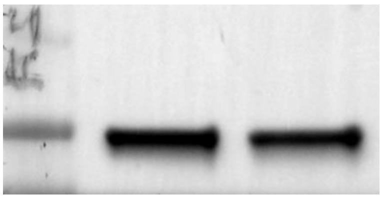

pSTAT3 S727 (MEN)

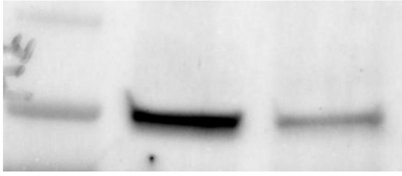

STAT3 for pSTAT3 S727 (MEN)

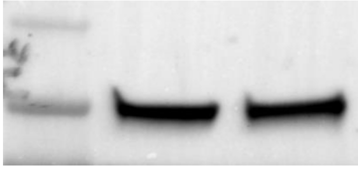

pSTAT6 Y641 (MEN)

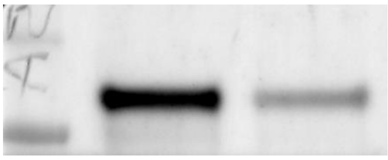

STAT6 (MEN)

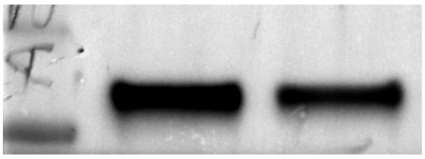

Related to Figure 3A (continued)

pCREB1 S133 (PIM447)

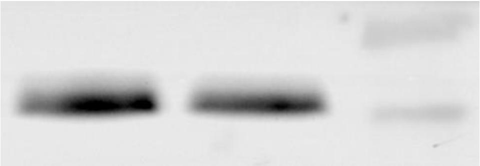

CREB1 (PIM447)

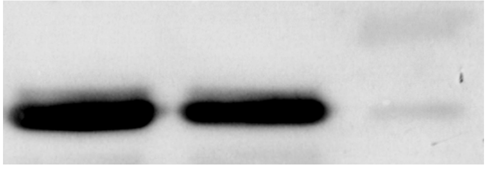

pSTAT3 Y705 (PIM447)

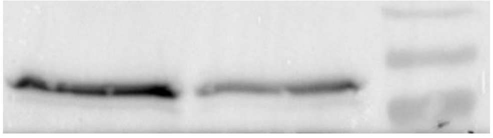

STAT3 for pSTAT3 Y705 (PIM447)

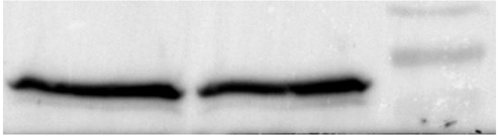

pSTAT3 S727 (PIM447)

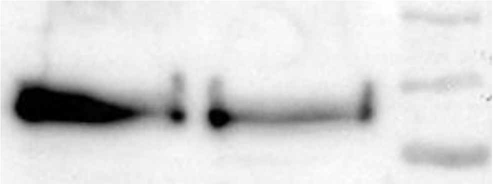

STAT3 for pSTAT3 S727 (PIM447)

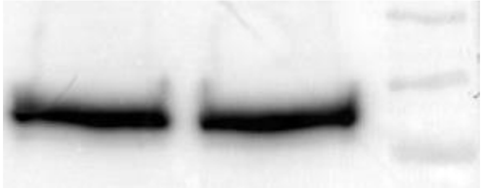

pSTAT6 Y641 (PIM447)

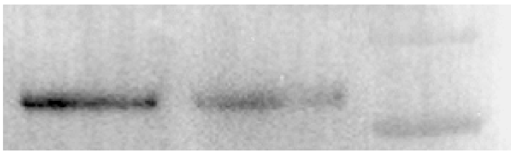

STAT6 (PIM447)

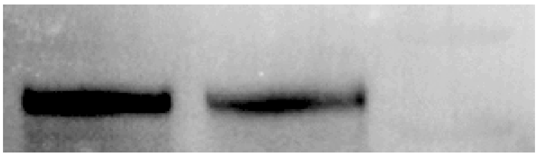

Related to Figure 4E

pCREB1 S133

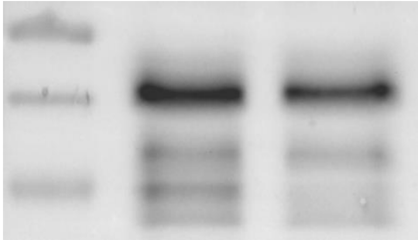

CREB1

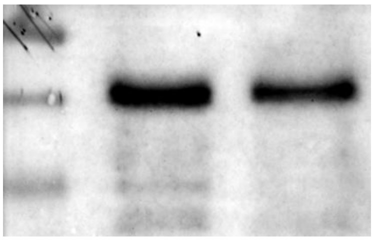

pSTAT3 Y705

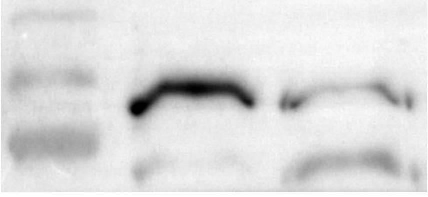

STAT3 for pSTAT3 Y705

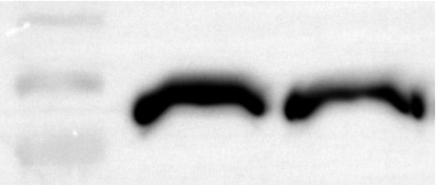

pSTAT3 S727

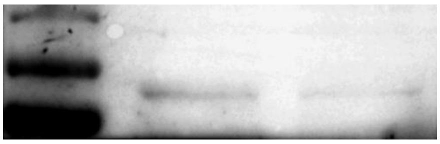

STAT3 for pSTAT3 S727

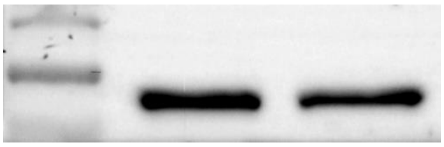

pSTAT6 Y641

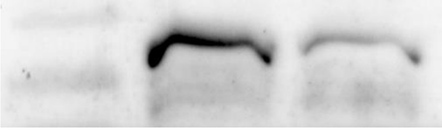

STAT6

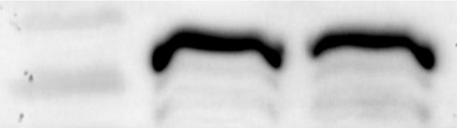

Related to Figure 4G

IL4I1

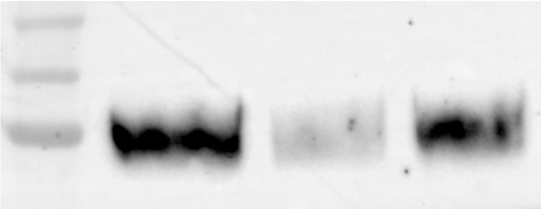

GAPDH

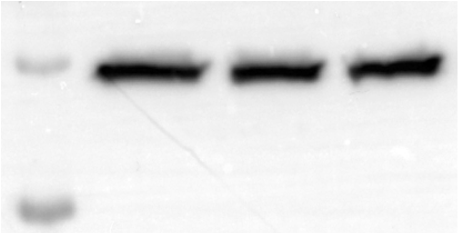

Related to Figure 4H

IDO1

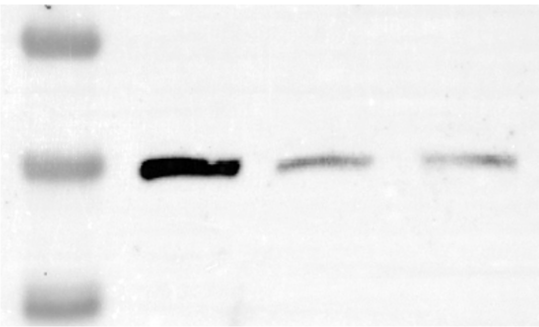

GAPDH

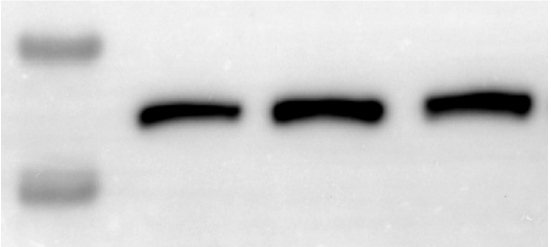

Related to supplemental Figure 3D

pCREB1 S133

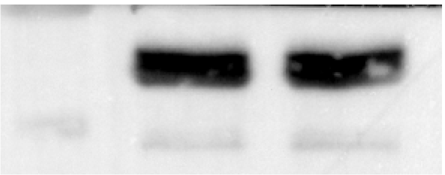

CREB1

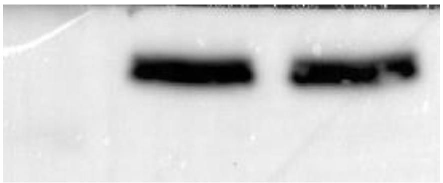

pSTAT3 Y705

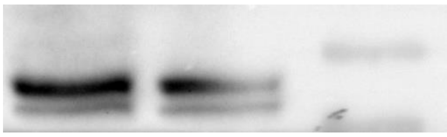

STAT3 for pSTAT3 Y705

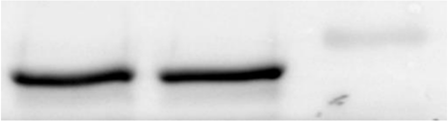

pSTAT3 S727

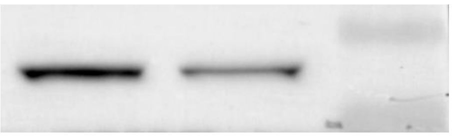

STAT3 for pSTAT3 S727

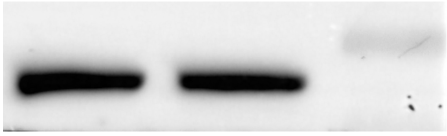

pSTAT6 Y641

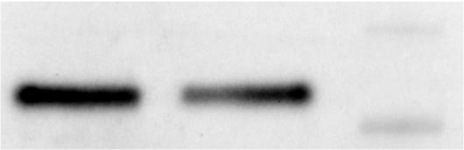

STAT6

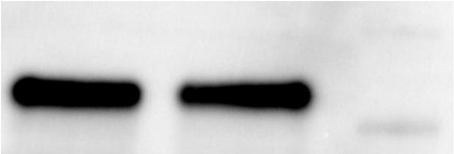

Related to supplemental Figure 3E

PIM1

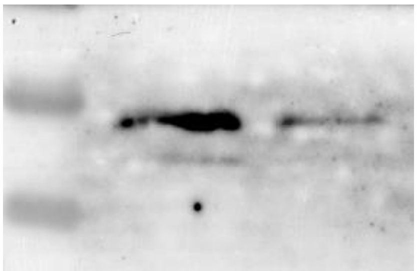

GAPDH for PIM1

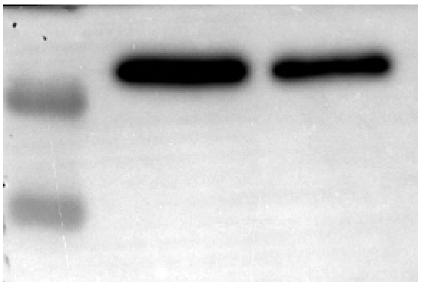

PIM2

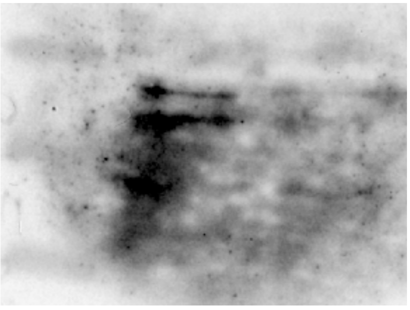

GAPDH for PIM2

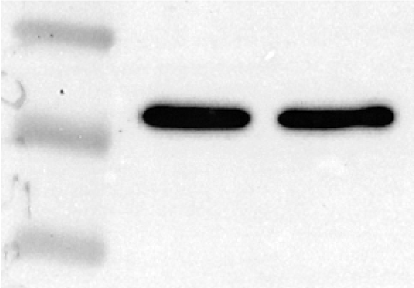

PIM3

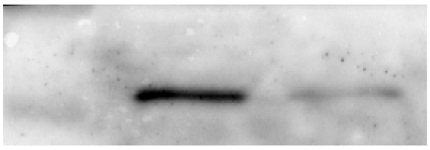

GAPDH for PIM3

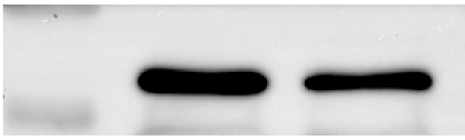

pCREB1 S133

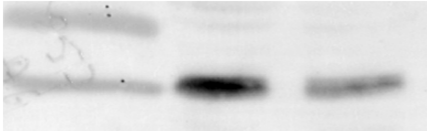

CREB1

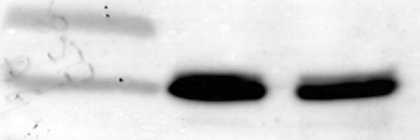

Related to supplemental Figure 3E (continued)

pSTAT3 Y705

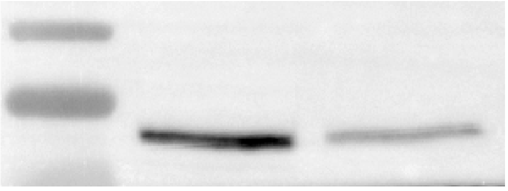

STAT3 for pSTAT3 Y705

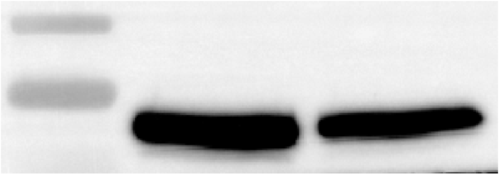

pSTAT3 S727

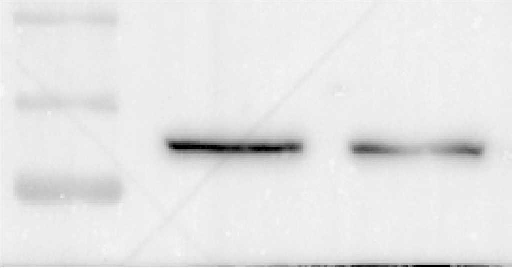

STAT3 for pSTAT3 S727

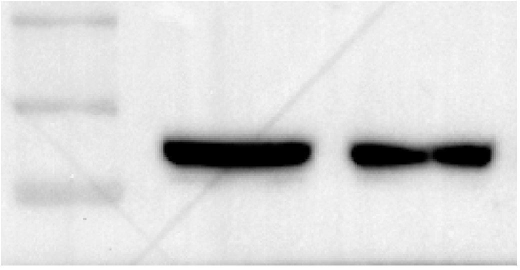

pSTAT6 Y641*

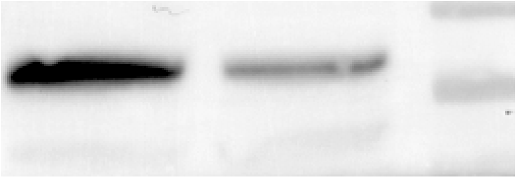

STAT6*

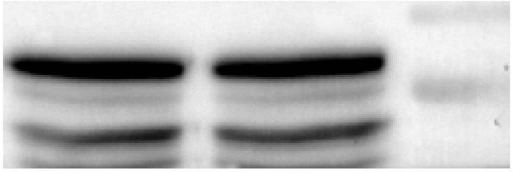

GAPDH for pSTAT6 Y641

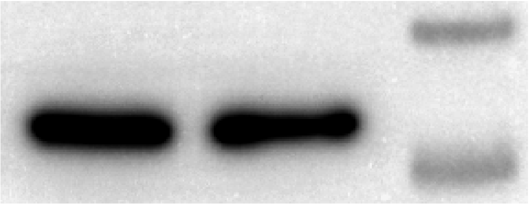

GAPDH for STAT6

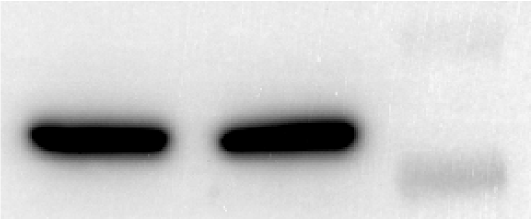

* - pSTAT6 and STAT6 were developed from separate membranes using the same protein lysates. Corresponding blots with loading control (GAPDH) are provided below.
